# Race, Ethnicity, and Complications Following Gender-Affirming Surgery: A National Surgical Quality Improvement Program (NSQIP) Analysis

**DOI:** 10.7759/cureus.96699

**Published:** 2025-11-12

**Authors:** Yu Jui Kung, Bashar Hassan, Calvin Schuster, Claudia Taccheri, Essie Ghafoor, Mona Ascha, Fan Liang

**Affiliations:** 1 Center for Transgender and Gender Expansive Health, Johns Hopkins University School of Medicine, Baltimore, USA; 2 Plastic and Reconstructive Surgery, Northwestern University Feinberg School of Medicine, Chicago, USA

**Keywords:** gender-affirming surgery, national surgical quality improvement program (nsqip), postoperative complications, racial and ethnic disparities, transgender and non-binary

## Abstract

Background: Transgender and non-binary (TGNB) individuals face disproportionately limited access to gender-affirming surgery (GAS) due to systemic inequities. This study examines how these inequities, such as race and ethnicity, impact the utilization of GAS and associated surgical complications across different GAS subtypes.

Methods: Using the American College of Surgeons National Surgical Quality Improvement Program (ACS-NSQIP) database, we retrospectively reviewed TGNB individuals who underwent GAS from 2012 to 2021. The primary outcome was the incidence of major complications (e.g., unplanned reoperation and readmission) and minor complications (e.g., wound complications) within 30 days postoperatively. Bivariate and multivariable tests were used to compare the frequency of GAS and complications across racial and ethnic groups.

Results: Among n = 6013 TGNB individuals, most were White (n = 3994, 66.4%), followed by Black (n = 880, 14.6%) and Hispanic (n = 788, 13.1%). Chest masculinization surgery was the most common procedure (n = 2395, 39.8%), followed by genital masculinization surgery (n = 1298, 21.6%), genital feminization surgery (n = 958, 15.9%), chest feminization surgery (n = 843, 14%), and facial feminization surgery (n = 396, 6.6%). White individuals were significantly more likely to undergo chest and genital masculinization surgery than Hispanic and Black individuals. Black individuals undergoing chest masculinization surgery had a higher frequency of unplanned reoperation (n = 14, 4.5%; n = 28, 1.7%; p = 0.042) and readmission (n = 7, 2.3%; n = 8, 0.5%; p = 0.048) compared with White individuals. For genital feminization surgery, Hispanic and Black individuals experienced significantly higher frequencies of wound disruption than White individuals (n = 12, 9.8%; n = 12, 8.7%; n = 21, 3.3%; p = 0.006). No significant disparities were observed for chest feminization, genital masculinization, or facial feminization surgeries.

Conclusion: Racial and ethnic disparities exist in surgical utilization and complications among TGNB individuals, highlighting the need for additional research and possibly targeted interventions to address these inequities.

## Introduction

According to the Centers for Disease Control and Prevention (CDC)’s Behavioral Risk Factor Surveillance System (BRFSS) and Youth Risk Behavior Survey (YRBS), over 1.6 million individuals, ages 13 and above, identify as transgender and non-binary (TGNB) [1]. This community faces significant challenges associated with gender dysphoria, including heightened rates of mental health conditions such as anxiety, depression, and suicidality-conditions often exacerbated by societal stigma, discrimination, and systemic inequities [2]. For many TGNB individuals, gender-affirming care (GAC) helps address the incongruence between their gender identity and physical characteristics, which can alleviate gender dysphoria and improve mental health outcomes [2]. GAC includes hormone replacement therapy (HRT) and gender-affirming surgeries (GAS) such as chest surgery, genital surgery, head and neck surgery, and body contouring. For those who pursue it, GAC is associated with significantly reduced rates of depression, suicidality, and gender dysphoria, as well as enhanced quality of life [2].

Despite its benefits, access to GAC remains limited, particularly for racial and ethnically marginalized TGNB individuals. Barriers such as financial constraints, gaps in insurance coverage, provider shortages, and pervasive discrimination within healthcare settings disproportionately impact these individuals, compounding the challenges faced by the broader TGNB community [3,4]. The intersectionality of being TGNB and a member of a racially or ethnically marginalized group magnifies the effects of structural racism, socioeconomic disadvantages, and medical discrimination, creating additional barriers to care [5].

Racial and ethnic disparities in surgical complications are well-documented across various medical fields, including oncologic, orthopedic, and cardiovascular surgeries. Black and Hispanic individuals consistently experience higher rates of complications, prolonged hospital stays, and unplanned reoperations, even after controlling for access and socioeconomic factors [6-9]. Although research has begun to examine disparities in TGNB populations undergoing GAS, these studies often do not delve into the nuances of how such disparities manifest in surgical complications across specific surgical subtypes to comprehensively evaluate the compounded barriers faced by these individuals [10,11]. Existing studies on GAS complications have primarily grouped procedures into broad categories, such as masculinizing or feminizing, without accounting for the wide variability in surgical techniques, risks, and complications associated with specific procedures (e.g., chest, genital, and facial surgeries) [10,11].

To address these gaps, this study leverages the American College of Surgeons National Surgical Quality Improvement Program (ACS-NSQIP) database to examine racial and ethnic disparities in the utilization of GAS and associated surgical complications, with a focus on stratifying specific surgical subtypes to gain a more detailed understanding of these inequities. This represents the first comprehensive analysis of GAS stratified by surgical subtype. The observed associations highlight emerging trends that may guide future investigations aimed at understanding and mitigating these disparities. The objective of this study is to evaluate racial and ethnic disparities in the utilization and 30-day postoperative complications of GAS among TGNB individuals, stratified by surgical subtype, to identify procedure-specific inequities that may inform efforts to advance equitable surgical care.

## Materials and methods

Population source

We queried the ACS-NSQIP database from 2012 to 2021. The ACS-NSQIP collects risk-adjusted, prospectively gathered data on patient demographics, comorbidities, operative characteristics, and 30-day postoperative outcomes to improve the quality of surgical care [12]. Trained surgical clinical reviewers at more than 750 voluntarily participating hospitals, including critical access, community, and large academic teaching hospitals across the United States, Canada, and 11 other countries, collect these data [12]. This study utilized de-identified patient data with an exempt approval obtained from the Johns Hopkins Institutional Review Board (IRB).

Study population

TGNB individuals within the ACS-NSQIP database were first identified using International Classification of Diseases (ICD)-9 and ICD-10 codes for gender dysphoria. These individuals were analyzed overall and across six subtypes of gender-affirming procedures: chest surgery in general, chest masculinization surgery, chest feminization surgery, genital masculinization surgery, genital feminization surgery, and facial feminization surgery. Patient selection and stratification were based on ICD and current procedural terminology (CPT) codes detailed in Table 1.

**Table 1:** ICD-9, ICD-10, and CPT codes used for patient selection and stratification ICD: International Classification of Diseases; CPT: current procedural terminology; CMS: chest masculinization surgery; CFS: chest feminization surgery ICD and CPT codes were selected by the study team based on clinical relevance and coding guidelines [13]

Code category	ICD-9, ICD-10, and CPT codes
ICD-9 codes for gender dysphoria	302.50, 302.51, 302.52, 302.53, 302.6, 302.85
ICD-10 codes for gender dysphoria	F64.0, F64.1, F64.2, F64.8, F64.9
CPT codes for CMS	15200, 15201, 19300, 19301, 19303, 19304, 19318, 19350
CPT codes for CFS	19324, 19325, 19340, 19342, 19366, 19370, 19371
CPT codes for genital masculinization surgery	15374, 15378, 15757, 15758, 53410, 53430, 53520, 55980, 56620, 57106, 57107, 57110, 57111, 58150, 58260, 58262, 58263, 58270, 58290, 58542, 58552, 58571, 58572, 58573, 58661
CPT codes for genital feminization surgery	53415, 54125, 54520, 54522, 54530, 55970, 56805, 57291, 57292, 57335
CPT codes for facial feminization surgery	21120, 21121, 21137, 21138, 21139, 21172, 21175, 21179, 21180, 21193, 21198, 21206, 21208, 21209, 21256, 31599, 31750, 31899

Individuals were categorized into six racial and ethnic categories per the U.S. Office of Management and Budget (OMB) 1997 standards: Hispanic, Non-Hispanic American Indian and Alaska Native, Non-Hispanic Asian, Non-Hispanic Black or African American, Non-Hispanic Native Hawaiian and Other Pacific Islander, and Non-Hispanic White [14]. The words “Hispanic” and “Latinx” were both used to refer to the same ethnic group. For the purposes of this study, American Indian or Alaska Native refers to Non-Hispanic American Indian and Alaska Native, Asian refers to Non-Hispanic Asian, Black refers to Non-Hispanic Black or African American, Native Hawaiian and Other Pacific Islander refers to Non-Hispanic Native Hawaiian and Other Pacific Islander, and White refers to Non-Hispanic White. Race and ethnicity classifications in the ACS-NSQIP are derived from administrative and clinical documentation at participating hospitals.

Covariates

Data on patient demographics, comorbidities, and surgical characteristics were collected, including age, body mass index (BMI), total operating time, hypertension, diabetes mellitus, smoking history within one year of surgery, current steroid use for chronic condition, preoperative dialysis, preoperative dyspnea, history of chronic obstructive pulmonary disease, history of a bleeding disorder, transfusion of one or more units of packed red blood cells within 72 hours preoperatively, >10% body weight loss within six months preoperatively, functional health status, surgical specialty, elective versus non-elective surgery, wound classification, and American Society of Anesthesiologists (ASA) Classification.

Outcomes

Our primary outcome was the incidence of major and minor complications within 30 days postoperatively. Major complications included cerebrovascular accident (CVA)/stroke, myocardial infarction (MI), cardiac arrest requiring cardiopulmonary resuscitation (CPR), sepsis, septic shock, pulmonary embolism (PE), deep venous thrombosis (DVT), acute renal failure, bleeding requiring transfusion, unplanned reoperation, unplanned readmission, unplanned intubation, ventilator-assisted respirations for >48 hours, and prolonged hospital stay (>30 days). Minor complications included pneumonia, wound disruption, surgical site infection (SSI), urinary tract infection (UTI), and *Clostridioides difficile* (C. diff) infection. Variable definitions followed ACS-NSQIP standards [15]. 

Statistical analysis

Patient demographics, comorbidities, surgical characteristics, major, and minor complications were compared across racial and ethnic groups using bivariate analysis. Descriptive statistics were calculated for the overall cohort and for each racial and ethnic group. Normally distributed continuous variables were presented as mean ± standard deviation (SD) and compared using the analysis of variance (ANOVA) test. Non-normally distributed continuous variables were presented as median (interquartile range, IQR) and compared using the Kruskal-Wallis test. Categorical variables were summarized as counts and percentages and compared using the chi-square or Fisher’s exact test as appropriate.

Pairwise comparisons among racial and ethnic groups were analyzed using Bonferroni post hoc analysis. Covariates with a p-value of <0.2 in bivariate analysis were included in multivariable logistic regression models to examine the potential association between race/ethnicity and the odds of complications. Adjusted odds ratios (aORs) and 95% confidence intervals (CIs) quantified the strength of these associations. A two-sided significance level was set at p < 0.05. All analyses were performed using IBM SPSS Statistics for Windows, Version 29 (Released 2022; IBM Corp., Armonk, New York, United States) [16]. 

## Results

Between 2012 and 2021, the ACS-NSQIP database identified 7508 individuals with gender dysphoria who underwent GAS. Those with unknown ethnicity (n = 1111, 14.79%) or unknown race (n = 384, 5.11%) were excluded, resulting in a final cohort of 6013 TGNB individuals eligible for analysis. Surgical outcomes were stratified by specific surgical subtypes and racial/ethnic categories to investigate disparities in surgical utilization and postoperative complications.

Patient demographics and surgical characteristics

Table 2 summarizes the frequency of GAS procedures by racial and ethnic groups. White individuals accounted for the majority (n = 3994, 66.4%) of the cohort, followed by Black individuals (n = 880, 14.6%) and the other groups. Chest masculinization surgery was the most commonly performed procedure (n = 2395, 39.8%), followed by genital masculinization (n = 1298, 21.6%) and feminization (n = 958, 15.9%) surgeries, chest feminization surgery (n = 843, 14%), and facial feminization surgery (n = 396, 6.6%). White individuals were significantly more likely to undergo chest masculinization surgery compared with Hispanic and Black individuals (n = 1661, 41.6%; n = 276, 35.0%; n = 311, 35.3%; p <0.001). The same pattern was observed for genital masculinization surgery (n = 980, 24.5%; n = 109, 13.8%; n = 150, 17.0%; p < 0.001). However, chest feminization surgery utilization was disproportionately lower among White individuals compared with Hispanic and Black individuals (n = 399, 10.0%; n = 195, 24.7%; n = 195, 22.2%; p < 0.001). Native Hawaiian or Pacific Islander individuals were more likely to undergo facial feminization surgery compared to Hispanic (n = 6, 28.6%; n = 71, 9.0%; p < 0.001) and White individuals (n = 6, 28.6%; n = 237, 5.9%; p < 0.001).

**Table 2:** Subtypes of gender-affirming surgeries stratified by racial and ethnic groups Frequency data are reported as No. (%). P-values were calculated using Fisher’s exact test due to low expected cell counts across groups. Racial and ethnic categories that do not share the same subscript letter differ significantly at p < .05

Type of gender-affirming surgery	Hispanic (n = 788)	Non-Hispanic American Indian or Alaska Native (n = 38)	Non-Hispanic Asian (n = 292)	Non-Hispanic Black or African American (n = 880)	Non-Hispanic Native Hawaiian or Pacific Islander (n = 21)	Non-Hispanic White (n = 3994)	Total (n = 6013)
Chest masculinization surgery	276 (35.0)_a_	14 (36.8)_a,b_	124 (42.5)_a,b_	311 (35.3)_a_	9 (42.9)_a,b_	1661 (41.6)_b_	2395 (39.8)
Chest feminization surgery	195 (24.7)_a_	6 (15.8)_a,b,c_	46 (15.8)_c_	195 (22.2)_a,c_	2 (9.5)_a,b,c_	399 (10.0)_b_	843 (14.0)
Genital masculinization surgery	109 (13.8)_a_	9 (23.7)_a,b_	49 (16.8)_a_	150 (17.0)_a_	1 (4.8)_a,b_	980 (24.5)_b_	1298 (21.6)
Genital feminization surgery	123 (15.6)_a_	8 (21.1)_a_	45 (15.4)_a_	138 (15.7)_a_	3 (14.3)_a_	641 (16.0)_a_	958 (15.9)
Facial feminization surgery	71 (9.0)_a_	1 (2.6)_a,b_	22 (7.5)_a,b_	59 (6.7)_a,b_	6 (28.6)_c_	237 (5.9)_b_	396 (6.6)
Other/unclear	14 (1.8)_a_	0 (0.0)_a_	6 (2.1)_a_	27 (3.1)_a_	0 (0.0)_a_	76 (1.9)_a_	123 (2.0)

Table 3 compares demographics, comorbidities, and surgical characteristics across racial and ethnic groups. Black and White individuals who underwent GAS were significantly older than Asian individuals (median (IQR) age: 30 (25-36), 28 (23-36), 27 (23-34) years; p = 0.002). Black individuals had significantly greater BMI at surgery compared with White and Hispanic individuals, who had significantly greater BMI compared with Asian individuals (median (IQR) BMI: 28.2 (24.1-33.1), 26.7 (23.2-31.9), 27.2 (23.6-31.4), 24.4 (21.7-27.9) kg/m2; p < 0.001). Hypertension was most prevalent among Black individuals compared with Hispanic, White, and Asian individuals (n = 87, 9.9%; n = 37, 4.7%; n = 278, 7.0%; n = 8, 2.7%; p < 0.001). Additionally, Black individuals were significantly more likely to be smokers compared with Hispanic, White, and Asian individuals (n = 176, 20%; n = 106, 13.5%; n = 479, 12.0%; n = 31, 10.6%; p < 0.001). Finally, ASA classifications were significantly higher among Black and White individuals compared with Asian individuals (p < 0.001). A similar comparative analysis of demographics, comorbidities, and surgical characteristics for individual GAS procedures is provided in Tables 4-8.

**Table 3:** Demographics, comorbidities, and surgical characteristics of patients who underwent any gender-affirming surgery across racial and ethnic groups IQR: interquartile range; BMI: body mass index; COPD: chronic obstructive pulmonary disorder; PRBC: packed red blood cells; ASA: American Society of Anesthesiologists Frequency data are reported as No. (%). Likelihood ratio chi-square test in logistic regression for surgical specialty: χ²(6) = 55.50. If chi-square test statistics are not provided, p-values were calculated using Fisher’s exact test due to low event counts. Racial and ethnic categories that do not share the same subscript letter “'a', b', 'c', etc.” differ significantly at p < .05

Racial and ethnic categories		Hispanic (n = 788)	Non-Hispanic American Indian or Alaska Native (n = 38)	Non-Hispanic Asian (n = 292)	Non-Hispanic Black or African American (n = 880)	Non-Hispanic Native Hawaiian or Pacific Islander (n = 21)	Non-Hispanic White (n = 3994)	P
Age, median (IQR), years		29 (24.0-37.0)_ab_	30.5 (23.0-37.0)_ab_	27 (23.0-33.8)_a_	30 (25.0-36.0)_b_	25 (21.0-33.5)_ab_	28 (23.0-36.0)_b_	.002
BMI, median (IQR), kg/m^2^		27.2 (23.6-31.4)_a_	27.1 (23.4-31.3)_abc_	24.4 (21.7-27.9)_b_	28.2 (24.1-33.1)_c_	27.3 (23.7-31.2)_abc_	26.7 (23.2-31.9)_a_	< .001
Operating time, median (IQR), minutes		139 (90.3-212.0)_a_	106 (74.5-194.8)_a_	138.5 (90.0-197.8)_a_	130 (88.0-201.0)_a_	107 (83.0-158.0)_a_	128 (88.0-190.0)_a_	.209
Surgical specialty	General surgery	26 (3.3)_a_	0 (0.0)_a_	10 (3.4)_a_	43 (4.9)_a_	0 (0.0)_a_	144 (3.6)_a_	< .001
	Gynecology	82 (10.4)_a_	8 (21.1)_ab_	34 (11.6)_a_	97 (11.0)_a_	0 (0.0)_ab_	811 (20.3)_ab_	
	Obstetric surgery	0 (0.0)_a_	0 (0.0)_a_	0 (0.0)_a_	0 (0.0)_a_	0 (0.0)_a_	3 (0.1)_a_	
	Orthopedic surgery	0 (0.0)_a_	0 (0.0)_a_	0 (0.0)_a_	0 (0.0)_a_	0 (0.0)_a_	2 (0.1)_a_	
	Otolaryngology (ENT)	54 (6.9)_a_	1 (2.6)_abcd_	21 (7.2)_ad_	21 (2.4)_c_	5 (23.8)_bd_	181 (4.5)_ac_	
	Plastic surgery	590 (74.9)_a_	24 (63.2)_ab_	210 (71.9)_ab_	655 (74.4)_a_	13 (61.9)_ab_	2549 (63.8)_b_	
	Urology	36 (4.6)_a_	5 (13.2)_ab_	17 (5.8)_ab_	64 (7.3)_ab_	3 (14.3)_ab_	304 (7.6)_b_	
Elective surgery		785 (99.6)_a_	38 (100.0)_a_	292 (100.0)_a_	880 (100.0)_a_	21 (100.0)_a_	3983 (99.7)_a_	.404
Hypertension requiring medication		37 (4.7)_a_	3 (7.9)_ab_	8 (2.7)_a_	87 (9.9)_b_	1 (4.8)_ab_	278 (7.0)_a_	< .001
Diabetes	On insulin	9 (1.1)_a_	1 (2.6)_a_	2 (0.7)_a_	5 (0.6)_a_	0 (0.0)_a_	48 (1.2)_a_	.689
	On oral agents	11 (1.4)_a_	1 (2.6)_a_	4 (1.4)_a_	17 (1.9)_a_	0 (0.0)_a_	76 (1.9)_a_	
Dyspnea	Moderate exertion	216 (27.4)_a_	8 (21.1)_a_	79 (27.1)_a_	213 (24.2)_a_	10 (47.6)_a_	1075 (26.9)_a_	.316
	At rest	0 (0.0)_a_	0 (0.0)_a_	0 (0.0)_a_	0 (0.0)_a_	0 (0.0)_a_	3 (0.1)_a_	
Current smoker within 1 year prior to surgery		106 (13.5)_a_	5 (13.2)_ab_	31 (10.6)_a_	176 (20.0)_b_	4 (19.0)_ab_	479 (12.0)_a_	< .001
Functional health status prior to surgery		786 (99.7)_a_	38 (100.0)_a_	292 (100.0)_a_	878 (99.8)_a_	21 (100.0)_a_	3977 (99.6)_a_	.815
History of severe COPD		1 (0.1)_a_	0 (0.0)_a_	0 (0.0)_a_	0 (0.0)_a_	0 (0.0)_a_	12 (0.3)_a_	.469
Preoperative dialysis		1 (0.1)_a_	0 (0.0)_a_	0 (0.0)_a_	0 (0.0)_a_	0 (0.0)_a_	0 (0.0)_a_	.189
Steroid use for chronic condition		3 (0.4)_a_	0 (0.0)_a_	1 (0.3)_a_	6 (0.7)_a_	0 (0.0)_a_	56 (1.4)_a_	.070
>10% body weight loss within 6 months prior to surgery		216 (27.4)_a_	8 (21.1)_a_	78 (26.7)_a_	214 (24.3)_a_	10 (47.6)_a_	1069 (26.8)_a_	.173
Bleeding disorder		2 (0.3)_ab_	1 (2.6)_b_	2 (0.7)_ab_	1 (0.1)_a_	0 (0.0)_ab_	29 (0.7)_ab_	.061
Transfusion >/= 1 units of PRBCs in 72 Hours prior to surgery		0 (0.0)_a_	0 (0.0)_a_	0 (0.0)_a_	1 (0.1)_a_	0 (0.0)_a_	0 (0.0)_a_	.336
Wound Classification	Clean	607 (77.0)_a_	26 (68.4)_ab_	229 (78.4)_ab_	684 (77.7)_a_	18 (85.7)_ab_	2871 (71.9)_b_	< .001
	Clean/contaminated	177 (22.5)_a_	12 (31.6)_ab_	63 (21.6)_ab_	193 (21.9)_a_	3 (14.3)_ab_	1114 (27.9)_b_	
	Contaminated	4 (0.5)_a_	0 (0.0)_a_	0 (0.0)_a_	2 (0.2)_a_	0 (0.0)_a_	8 (0.2)_a_	
	Dirty/infected	0 (0.0)_a_	0 (0.0)_a_	0 (0.0)_a_	1 (0.1)_a_	0 (0.0)_a_	1 (0.0)_a_	
ASA Classification	I	189 (24.0)_a_	10 (26.3)_ab_	113 (38.7)_b_	200 (22.7)_a_	7 (33.3)_ab_	1067 (26.7)_a_	< .001
	II	554 (70.3)_a_	26 (68.4)_ab_	172 (58.9)_b_	602 (68.4)_a_	13 (61.9)_ab_	2607 (65.3)_ab_	
	III	42 (5.3)_ab_	2 (5.3)_ab_	7 (2.4)_b_	78 (8.9)_a_	1 (4.8)_ab_	317 (7.9)_a_	
	IV	2 (0.3)_a_	0 (0.0)_a_	0 (0.0)_a_	0 (0.0)_a_	0 (0.0)_a_	3 (0.1)_a_	
	None assigned	1 (0.1)_a_	0 (0.0)_a_	0 (0.0)_a_	0 (0.0)_a_	0 (0.0)_a_	0 (0.0)_a_	

**Table 4:** Demographics, comorbidities, and surgical characteristics of patients who underwent chest masculinization surgery across racial and ethnic groups IQR: interquartile range; BMI: body mass index; COPD: chronic obstructive pulmonary disorder; PRBC: packed red blood cells; ASA: American Society of Anesthesiologists Frequency data are reported as No. (%). p-values were calculated using Fisher’s exact test due to low expected cell counts across groups. Racial and ethnic categories that do not share the same subscript letter “'a', 'b', 'c', etc.” differ significantly at p < .05

Covariate		Hispanic (n = 276)	Non-Hispanic American Indian or Alaska Native (n = 14)	Non-Hispanic Asian (n = 124)	Non-Hispanic Black or African American (n = 311)	Non-Hispanic Native Hawaiian or Pacific Islander (n = 9)	Non-Hispanic White (n = 1661)	p
Age, median (IQR), years		24 (21.0-30.0)_a_	26 (20.5-31.8)_ab_	25 (21.0-30.0)_ab_	27 (23.0-32.0)_b_	23 (20.0-29.5)_ab_	25 (21.0-30.0)_a_	.013
BMI, median (IQR), kg/m^2^		27.4 (23.7-32.4)_a_	29 (26.2-35.7)_ab_	25.4 (22.5-28.8)_b_	28.3 (24.3-33.6)_a_	27.3 (23.7-31.7)_ab_	27.4 (23.5-32.5)_a_	< .001
Operating time, median (IQR), minutes		152 (119.0-189.8)_a_	125 (91.5-141.0)_ab_	142 (110.0-169.0)_ab_	147 (116.0-189.0)_a_	107 (98.5-138.0)_ab_	134 (105.5-170.0)_b_	< .001
Surgical specialty	General surgery	18 (6.5)_a_	0 (0.0)_a_	9 (7.3)_a_	26 (8.4)_a_	0 (0.0)_a_	127 (7.6)_a_	.940
	Gynecology	1 (0.4)_a_	0 (0.0)_a_	0 (0.0)_a_	0 (0.0)_a_	0 (0.0)_ab_	2 (0.1)_a_	
	Obstetric surgery	0 (0.0)_a_	0 (0.0)_a_	0 (0.0)_a_	0 (0.0)_a_	0 (0.0)_a_	1 (0.1)_a_	
	Plastic surgery	257 (93.1)_a_	14 (100.0)_a_	115 (92.7)_a_	285 (91.6)_a_	9 (100.0)_a_	1530 (92.1)_a_	
	Urology	0 (0.0)_a_	0 (0.0)_a_	0 (0.0)_a_	0 (0.0)_a_	0 (0.0)_a_	1 (0.1)_a_	
Elective surgery		276 (100.0)_a_	14 (100.0)_a_	124 (100.0)_a_	311 (100.0)_a_	9 (100.0)_a_	1657 (99.8)_a_	1.0
Hypertension requiring medication		8 (2.9)_a_	0 (0.0)_a_	1 (0.8)_a_	21 (6.8)_a_	0 (0.0)_a_	59 (3.6)_a_	.054
Diabetes	On insulin	3 (1.1)_a_	0 (0.0)_a_	0 (0.0)_a_	0 (0.0)_a_	0 (0.0)_a_	14 (0.8)_a_	.468
	On oral agents	4 (1.4)_a_	1 (7.1)_a_	1 (0.8)_a_	3 (1.0)_a_	0 (0.0)_a_	22 (1.3)_a_	
Dyspnea	Moderate exertion	87 (31.5)_a_	3 (21.4)_a_	32 (25.8)_a_	79 (25.4)_a_	4 (44.4)_a_	431 (25.9)_a_	.490
	At rest	0 (0.0)_a_	0 (0.0)_a_	0 (0.0)_a_	0 (0.0)_a_	0 (0.0)_a_	1 (0.1)_a_	
Current smoker within 1 year prior to surgery		30 (10.9)_a_	3 (21.4)_a_	13 (10.5)_a_	59 (19.0)_a_	2 (22.2)_a_	228 (13.7)_a_	.036
Functional health status prior to surgery		274 (99.3)_a_	14 (100.0)_a_	124 (100.0)_a_	311 (100.0)_a_	9 (100.0)_a_	1654 (99.6)_a_	.531
History of severe COPD		0 (0.0)_a_	0 (0.0)_a_	0 (0.0)_a_	0 (0.0)_a_	0 (0.0)_a_	2 (0.1)_a_	1.0
Preoperative dialysis		0 (0.0)_a_	0 (0.0)_a_	0 (0.0)_a_	0 (0.0)_a_	0 (0.0)_a_	0 (0.0)_a_	-
Steroid use for chronic condition		2 (0.7)_a_	0 (0.0)_a_	0 (0.0)_a_	3 (1.0)_a_	0 (0.0)_a_	25 (1.5)_a_	.652
>10% body weight loss within 6 months prior to surgery		87 (31.5)_a_	3 (21.4)_a_	32 (25.8)_a_	79 (25.4)_a_	4 (44.4)_a_	428 (25.8)_a_	.306
Bleeding disorder		1 (0.4)_a_	0 (0.0)_a_	0 (0.0)_a_	0 (0.0)_a_	0 (0.0)_a_	10 (0.6)_a_	.695
Transfusion ≥ 1 units of PRBCs in 72 hours prior to surgery		0 (0.0)_a_	0 (0.0)_a_	0 (0.0)_a_	0 (0.0)_a_	0 (0.0)_a_	0 (0.0)_a_	-
Wound classification	1-Clean	274 (99.3)_a_	14 (100.0)_a_	123 (99.2)_a_	311 (100.0)_a_	9 (100.0)_a_	1653 (99.5)_a_	.570
	2-Clean/contaminated	2 (0.7)_a_	0 (0.0)_a_	1 (0.8)_a_	0 (0.0)_a_	0 (0.0)_a_	7 (0.4)_a_	
	3-Contaminated	0 (0.0)_a_	0 (0.0)_a_	0 (0.0)_a_	0 (0.0)_a_	0 (0.0)_a_	0 (0.0)_a_	
	4-Dirty/infected	0 (0.0)_a_	0 (0.0)_a_	0 (0.0)_a_	0 (0.0)_a_	0 (0.0)_a_	1 (0.1)_a_	
ASA classification	I	77 (27.9)_a_	3 (21.4)_a_	52 (41.9)_a_	89 (28.6)_a_	3 (33.3)_a_	514 (30.9)_a_	.899
	II	187 (67.8)_a_	10 (71.4)_a_	69 (55.6)_a_	206 (66.2)_a_	5 (55.6)_a_	1051 (63.3)_a_	
	III	11 (4.0)_a_	1 (7.1)_a_	3 (2.4)_a_	16 (5.1)_a_	1 (11.1)_a_	94 (5.7)_a_	
	IV	1 (0.4)_a_	0 (0.0)_a_	0 (0.0)_a_	0 (0.0)_a_	0 (0.0)_a_	2 (0.1)_a_	

**Table 5:** Demographics, comorbidities, and surgical characteristics of patients who underwent chest feminization surgery across racial and ethnic groups IQR: interquartile range; BMI: body mass index; COPD: chronic obstructive pulmonary disorder; PRBC: packed red blood cells; ASA: American Society of Anesthesiologists Frequency data are reported as No. (%). P-values were calculated using Fisher’s exact test due to low expected cell counts across groups. Racial and ethnic categories that do not share the same subscript letter “'a', 'b', 'c', etc.” differ significantly at p < .05

Covariate		Hispanic (n = 195)	Non-Hispanic American Indian or Alaska Native (n = 6)	Non-Hispanic Asian (n = 46)	Non-Hispanic Black or African American (n = 195)	Non-Hispanic Native Hawaiian or Pacific Islander (n = 2)	Non-Hispanic White (n = 399)	P
Age, median (IQR), years		32 (25.0-40.0)_a_	31.5 (23.0-39.0)_ab_	32 (26.8-39.0)_a_	31 (27.0-39.0)_a_	38.5 (22.0-38.5)_ab_	37 (29.0-51.0)_b_	< .001
BMI, median (IQR), kg/m^2^		26.9 (23.6-32.2)_a_	26.7 (19.8-29.6)_ab_	23.1 (21.1-27.0)_b_	27.6 (24.0-31.9)_a_	22.3 (20.3-22.3)_ab_	26 (22.4-30.4)_b_	< .001
Operating time, median (IQR), minutes		86 (63.0-106.0)_a_	67.5 (58.8-153.0)_a_	80 (63.3-121.5)_a_	86 (70.0-115.0)_a_	77.5 (74.0-77.5)_a_	81 (62.0-112.0)_a_	.139
Surgical specialty	General surgery	4 (2.1)_ab_	0 (0.0)_ab_	1 (2.2)_ab_	10 (5.1)_b_	0 (0.0)_ab_	3 (0.8)_a_	.048
	Orthopedic surgery	0 (0.0)_a_	0 (0.0)_a_	0 (0.0)_a_	0 (0.0)_a_	0 (0.0)_a_	1 (0.3)_a_	
	Plastic surgery	191 (97.9)_ab_	6 (100.0)_ab_	45 (97.8)_ab_	185 (94.9)_b_	2 (100.0)_ab_	395 (99.0)_a_	
Elective surgery		194 (99.5)_a_	6 (100.0)_a_	46 (100.0)_a_	195 (100.0)_a_	2 (100.0)_a_	395 (99.0)_a_	.719
Hypertension requiring medication		13 (6.7)_a_	1 (16.7)_a_	4 (8.7)_a_	22 (11.3)_a_	0 (0.0)_a_	55 (13.8)_a_	.138
Diabetes	On insulin	0 (0.0)_a_	0 (0.0)_a_	1 (2.2)_a_	2 (1.0)_a_	0 (0.0)_a_	7 (1.8)_a_	.538
	On oral agents	4 (2.1)_a_	0 (0.0)_a_	1 (2.2)_a_	6 (3.1)_a_	0 (0.0)_a_	16 (4.0)_a_	
Dyspnea	Moderate exertion	56 (28.7)_ab_	2 (33.3)_ab_	10 (21.7)_ab_	42 (21.5)_b_	1 (50.0)_ab_	137 (34.3)_a_	.017
Current smoker within 1 year prior to surgery		40 (20.5)_ab_	0 (0.0)_ab_	8 (17.4)_ab_	55 (28.2)_b_	1 (50.0)_ab_	61 (15.3)_a_	.004
Functional health status prior to surgery		195 (100.0)_a_	6 (100.0)_a_	46 (100.0)_a_	194 (99.5)_a_	2 (100.0)_a_	399 (100.0)_a_	.527
History of severe COPD		1 (0.5)_a_	0 (0.0)_a_	0 (0.0)_a_	0 (0.0)_a_	0 (0.0)_a_	3 (0.8)_a_	.855
Preoperative dialysis		0 (0.0)_a_	0 (0.0)_a_	0 (0.0)_a_	0 (0.0)_a_	0 (0.0)_a_	0 (0.0)_a_	-
Steroid use for chronic condition		0 (0.0)_a_	0 (0.0)_a_	0 (0.0)_a_	1 (0.5)_a_	0 (0.0)_a_	3 (0.8)_a_	.855
>10% body weight loss within 6 months prior to surgery		56 (28.7)_ab_	2 (33.3)_ab_	10 (21.7)_ab_	43 (22.1)_b_	1 (50.0)_ab_	136 (34.1)_a_	.029
Bleeding disorder		0 (0.0)_a_	0 (0.0)_a_	0 (0.0)_a_	0 (0.0)_a_	0 (0.0)_a_	3 (0.8)_a_	.536
Transfusion ≥1 units of PRBCs in 72 hours prior to surgery		0 (0.0)_a_	0 (0.0)_a_	0 (0.0)_a_	0 (0.0)_a_	0 (0.0)_a_	0 (0.0)_a_	-
Wound classification	1-Clean	193 (99.0)_a_	5 (83.3)_b_	44 (95.7)_ab_	190 (97.4)_ab_	2 (100.0)_ab_	383 (96.0)_ab_	.055
	2-Clean/contaminated	2 (1.0)_a_	1 (16.7)_b_	2 (4.3)_ab_	3 (1.5)_ab_	0 (0.0)_ab_	16 (4.0)_ab_	
	3-Contaminated	0 (0.0)_a_	0 (0.0)_a_	0 (0.0)_a_	0 (0.0)_a_	0 (0.0)_a_	0 (0.0)_a_	
	4-Dirty/infected	0 (0.0)_a_	0 (0.0)_a_	0 (0.0)_a_	0 (0.0)_a_	0 (0.0)_a_	0 (0.0)_a_	
ASA classification	I	43 (22.1)_a_	2 (33.3)_a_	14 (30.4)_a_	35 (17.9)_a_	0 (0.0)_a_	86 (21.6)_a_	-
	II	141 (72.3)_a_	3 (50.0)_a_	30 (65.2)_a_	145 (74.4)_a_	2 (100.0)_a_	270 (67.7)_a_	
	III	10 (5.1)_a_	1 (16.7)_a_	2 (4.3)_a_	15 (7.7)_a_	0 (0.0)_a_	43 (10.8)_a_	
	None assigned	1 (0.5)_a_	0 (0.0)_a_	0 (0.0)_a_	0 (0.0)_a_	0 (0.0)_a_	0 (0.0)_a_	

**Table 6:** Demographics, comorbidities, and surgical characteristics of patients who underwent genital masculinization surgery across racial and ethnic groups IQR: interquartile range; BMI: body mass index; COPD: chronic obstructive pulmonary disorder; PRBC: packed red blood cells; ASA: American Society of Anesthesiologists Frequency data are reported as No. (%). Pearson’s chi-square test comparing race/ethnicity and surgical subspecialty: χ²(25) = 40.2, p = .028; Cramér’s V = 0.79. Pearson’s chi-square test comparing race/ethnicity and ASA Classification: χ²(10) = 21.598, p = .017; Cramér’s V = 0.091. If chi-square test statistics are not provided, p-values were calculated using Fisher’s exact test due to low event counts. Racial and ethnic categories that do not share the same subscript letter “'a', 'b', 'c', etc.” differ significantly at p < .05

Covariate		Hispanic (n = 109)	Non-Hispanic American Indian or Alaska Native (n = 9)	Non-Hispanic Asian (n = 49)	Non-Hispanic Black or African American (n = 150)	Non-Hispanic Native Hawaiian or Pacific Islander (n = 1)	Non-Hispanic White (n = 980)	p
Age, median (IQR), years		28 (24.0-36.0)	30 (26.0-37.0)	26 (23.0-34.0)	30 (25.0-37.3)	41 (41.0-41.0)	27 (23.0-34.0)	.168
BMI, median (IQR), kg/m^2^		28.3 (23.6-31.9)	21.7 (20.8-28.0)	25.2 (22.6-29.8)	29.7 (24.3-34.1)	30.8 (30.8-30.8)	27.6 (24.0-32.6)	.008
Operating time, median (IQR), minutes		134 (95.0-184.0)	109 (79.5-228.0)	130 (88.0-199.5)	143.5 (96.8-247.3)	137 (137.0-137.0)	120.5 (88.0-176.8)	.009
Surgical specialty	General surgery	1 (0.9)_a_	0 (0.0)_a_	0 (0.0)_a_	0 (0.0)_a_	0 (0.0)_a_	3 (0.3)_a_	.028
	Gynecology	81 (74.3)_ab_	8 (88.9)_ab_	34 (69.4)_ab_	96 (64.0)_b_	0 (0.0)_ab_	804 (82.0)_a_	
	Obstetric surgery	0 (0.0)_a_	0 (0.0)_a_	0 (0.0)_a_	0 (0.0)_a_	0 (0.0)_a_	2 (0.2)_a_	
	Orthopedic surgery	0 (0.0)_a_	0 (0.0)_a_	0 (0.0)_a_	0 (0.0)_a_	0 (0.0)_a_	1 (0.1)_a_	
	Plastic surgery	23 (21.1)_ab_	1 (11.1)_ab_	13 (26.5)_ab_	47 (31.3)_b_	1 (100.0)_ab_	145 (14.8)_a_	
	Urology	4 (3.7)_a_	0 (0.0)_a_	2 (4.1)_a_	7 (4.7)_a_	0 (0.0)_a_	25 (2.6)_a_	
Elective surgery		109 (100.0)_a_	9 (100.0)_a_	49 (100.0)_a_	150 (100.0)_a_	1 (100.0)_a_	977 (99.7)_a_	1.0
Hypertension requiring medication		6 (5.5)_a_	1 (11.1)_ab_	3 (6.1)_a_	17 (11.3)_ab_	1 (100.0)_b_	80 (8.2)_a_	.100
Diabetes	On insulin	1 (0.9)_a_	0 (0.0)_a_	0 (0.0)_a_	1 (0.7)_a_	0 (0.0)_a_	15 (1.5)_a_	.888
	On oral agents	0 (0.0)_a_	0 (0.0)_a_	1 (2.0)_a_	2 (1.3)_a_	0 (0.0)_a_	12 (1.2)_a_	
Dyspnea	Moderate exertion	27 (24.8)_a_	2 (22.2)_a_	15 (30.6)_a_	56 (37.3)_a_	0 (0.0)_a_	260 (26.5)_a_	.217
	At rest	0 (0.0)_a_	0 (0.0)_a_	0 (0.0)_a_	0 (0.0)_a_	0 (0.0)_a_	1 (0.1)_a_	
Current smoker within 1 year prior to surgery		17 (15.6)_a_	0 (0.0)_a_	6 (12.2)_a_	24 (16.0)_a_	0 (0.0)_a_	101 (10.3)_a_	.179
Functional health status prior to surgery		109 (100.0)_a_	9 (100.0)_a_	49 (100.0)_a_	150 (100.0)_a_	1 (100.0)_a_	976 (99.6)_a_	1.0
History of severe COPD		0 (0.0)_a_	0 (0.0)_a_	0 (0.0)_a_	0 (0.0)_a_	0 (0.0)_a_	1 (0.1)_a_	1.0
Preoperative dialysis		1 (0.9)_a_	0 (0.0)_ab_	0 (0.0)_ab_	0 (0.0)_ab_	0 (0.0)_ab_	0 (0.0)_b_	.129
Steroid use for chronic condition		1 (0.9)_a_	0 (0.0)_a_	1 (2.0)_a_	1 (0.7)_a_	0 (0.0)_a_	8 (0.8)_a_	.578
>10% body weight loss within 6 months prior to surgery		27 (24.8)_a_	2 (22.2)_a_	14 (28.6)_a_	56 (37.3)_a_	0 (0.0)_a_	260 (26.5)_a_	.118
Bleeding disorder		0 (0.0)_a_	0 (0.0)_a_	2 (4.1)_a_	0 (0.0)_a_	0 (0.0)_a_	9 (0.9)_a_	.140
Transfusion ≥1 unit of PRBCs in 72 hours prior to surgery		0 (0.0)_a_	0 (0.0)_a_	0 (0.0)_a_	1 (0.7)_a_	0 (0.0)_a_	0 (0.0)_a_	.245
Wound classification	1-Clean	38 (34.9)_ab_	2 (22.2)_ab_	20 (40.8)_ab_	75 (50.0)_b_	0 (0.0)_ab_	326 (33.3)_a_	.008
	2-Clean/contaminated	69 (63.3)_ab_	7 (77.8)_ab_	29 (59.2)_ab_	75 (50.0)_b_	1 (100.0)_ab_	649 (66.2)_a_	
	3-Contaminated	2 (1.8)_a_	0 (0.0)_a_	0 (0.0)_a_	0 (0.0)_a_	0 (0.0)_a_	5 (0.5)_a_	
ASA classification	I	26 (23.9)_a_	2 (22.2)_ab_	24 (49.0)_b_	37 (24.7)_a_	0 (0.0)_ab_	243 (24.8)_a_	.017
	II	77 (70.6)_a_	7 (77.8)_a_	24 (49.0)_a_	94 (62.7)_a_	1 (100.0)_a_	644 (65.7)_a_	
	III	6 (5.5)_a_	0 (0.0)_a_	1 (2.0)_a_	19 (12.7)_a_	0 (0.0)_a_	93 (9.5)_a_	

**Table 7:** Demographics, comorbidities, and surgical characteristics of patients who underwent genital feminization surgery across racial and ethnic groups IQR: interquartile range; BMI: body mass index; COPD: chronic obstructive pulmonary disorder; PRBC: packed red blood cells; ASA: American Society of Anesthesiologists Frequency data are reported as No. (%). Pearson’s chi-square test comparing race/ethnicity and surgical subspecialty: χ²(20) = 30.629, p = .06; Cramér’s V = 0.089. Pearson’s chi-square test comparing race/ethnicity and ASA Classification: χ²(15) = 17.660, p = .281; Cramér’s V = 0.078. If chi-square test statistics are not provided, p-values were calculated using Fisher’s exact test due to low event counts. Racial and ethnic categories that do not share the same subscript letter “'a', 'b', 'c', etc.” differ significantly at p < .05

Covariate		Hispanic (n = 123)	Non-Hispanic American Indian or Alaska Native (n = 8)	Non-Hispanic Asian (n = 45)	Non-Hispanic Black or African American (n = 138)	Non-Hispanic Native Hawaiian or Pacific Islander (n = 3)	Non-Hispanic White (n = 641)	p
Age, median (IQR), years		31 (27.0-42.0)_ab_	40 (30.0-48.5)_ab_	28 (25.0-36.0)_ab_	34.5 (28.0-46.0)_a_	27 -	34 (27.0-47.0)_b_	.013
BMI, median (IQR), kg/m^2^		26.6 (23.5-29.1)_abc_	28.8 (25.5-33.6)_abc_	22.5 (20.4-26.2)_a_	27.6 (24.5-30.8)_b_	31.6 -	25.7 (22.2-29.8)_c_	< .001
Operating time, median (IQR), minutes		256 (112.0-313.0)_a_	96.5 (44.0-256.0)_ab_	285 (175.5- 363.0)_a_	197 (59.8-307.5)_ab_	32 -	169 (52.0-292.0)_b_	< .001
Surgical specialty	General surgery	1 (0.8)_a_	0 (0.0)_a_	0 (0.0)_a_	5 (3.6)_a_	0 (0.0)_a_	8 (1.2)_a_	.060
	Gynecology	0 (0.0)_a_	0 (0.0)_a_	0 (0.0)_a_	0 (0.0)_a_	0 (0.0)_a_	2 (0.3)_a_	
	Otolaryngology (ENT)	0 (0.0)_a_	0 (0.0)_a_	0 (0.0)_a_	0 (0.0)_a_	0 (0.0)_a_	1 (0.2)_a_	
	Plastic surgery	94 (76.4)_a_	3 (37.5)_ab_	32 (71.1)_ab_	78 (56.6)_b_	0 (0.0)_b_	372 (58.0)_b_	
	Urology	28 (22.8)_a_	5 (62.5)_ab_	13 (28.9)_ab_	55 (39.9)_b_	3 (100.0)_b_	258 (40.2)_b_	
Elective surgery		122 (99.2)_a_	8 (100.0)_a_	45 (100.0)_a_	138 (100.0)_a_	3 (100.0)_a_	641 (100.0)_a_	.187
Hypertension requiring medication		5 (4.1)_a_	1 (12.5)_a_	0 (0.0)_a_	19 (13.8)_a_	0 (0.0)_a_	64 (10.0)_a_	.011
Diabetes	On insulin	4 (3.3)_a_	1 (12.5)_a_	1 (2.2)_a_	2 (1.4)_a_	0 (0.0)_a_	9 (1.4)_a_	.269
	On oral agents	1 (0.8)_a_	0 (0.0)_a_	1 (2.2)_a_	6 (4.3)_a_	0 (0.0)_a_	17 (2.7)_a_	
Dyspnea	Moderate exertion	13 (10.6)_a_	1 (12.5)_abc_	12 (26.7)_abc_	17 (12.3)_ac_	2 (66.7)_bc_	164 (25.6)_b_	< .001
	At rest	0 (0.0)_a_	0 (0.0)_a_	0 (0.0)_a_	0 (0.0)_a_	0 (0.0)_a_	1 (0.2)_a_	
Current smoker within 1 year prior to surgery		15 (12.2)_a_	1 (12.5)_a_	2 (4.4)_a_	25 (18.1)_a_	1 (33.3)_a_	65 (10.1)_a_	.040
Functional health status prior to surgery		123 (100.0)_a_	8 (100.0)_a_	45 (100.0)_a_	137 (99.3)_a_	3 (100.0)_a_	635 (99.1)_a_	.895
History of severe COPD		0 (0.0)_a_	0 (0.0)_a_	0 (0.0)_a_	0 (0.0)_a_	0 (0.0)_a_	4 (0.6)_a_	1.0
Preoperative dialysis		0 (0.0)_a_	0 (0.0)_a_	0 (0.0)_a_	0 (0.0)_a_	0 (0.0)_a_	0 (0.0)_a_	-
Steroid use for chronic condition		0 (0.0)_a_	0 (0.0)_a_	0 (0.0)_a_	1 (0.7)_a_	0 (0.0)_a_	16 (2.5)_a_	.334
>10% body weight loss within 6 months prior to surgery		13 (10.6)_a_	1 (12.5)_abc_	12 (26.7)_abc_	17 (12.3)_ac_	2 (66.7)_bc_	162 (25.3)_b_	< .001
Bleeding disorder		1 (0.8)_ab_	1 (12.5)_b_	0 (0.0)_ab_	0 (0.0)_a_	0 (0.0)_ab_	5 (0.8)_a_	.136
Transfusion ≥1 units of PRBCs in 72 hours prior to surgery		0 (0.0)_a_	0 (0.0)_a_	0 (0.0)_a_	0 (0.0)_a_	0 (0.0)_a_	0 (0.0)_a_	-
Wound classification	1-Clean	38 (30.9)_a_	4 (50.0)_ab_	19 (42.2)_ab_	49 (35.5)_ab_	2 (66.7)_ab_	295 (46.0)_b_	.037
	2-Clean/contaminated	84 (68.3)_a_	4 (50.0)_ab_	26 (57.8)_ab_	88 (63.8)_ab_	1 (33.3)_ab_	344 (53.7)_b_	
	3-Contaminated	1 (0.8)_a_	0 (0.0)_a_	0 (0.0)_a_	1 (0.7)_a_	0 (0.0)_a_	2 (0.3)_a_	
ASA classification	I	18 (14.6)_a_	3 (37.5)_a_	14 (31.1)_a_	23 (16.7)_a_	1 (33.3)_a_	142 (22.2)_a_	.281
	II	99 (80.5)_a_	5 (62.5)_a_	30 (66.7)_a_	99 (71.7)_a_	2 (66.7)_a_	435 (67.9)_a_	
	III	6 (4.9)_a_	0 (0.0)_a_	1 (2.2)_a_	16 (11.6)_a_	0 (0.0)_a_	63 (9.8)_a_	
	IV	0 (0.0)_a_	0 (0.0)_a_	0 (0.0)_a_	0 (0.0)_a_	0 (0.0)_a_	1 (0.2)_a_	

**Table 8:** Demographics, comorbidities, and surgical characteristics of patients who underwent facial feminization surgery across racial and ethnic groups IQR: interquartile range; BMI: body mass index; COPD: chronic obstructive pulmonary disorder; PRBC: packed red blood cells; ASA: American Society of Anesthesiologists Frequency data are reported as No. (%). P-values were calculated using Fisher’s exact test due to low expected cell counts across groups. Racial and ethnic categories that do not share the same subscript letter “'a', 'b', 'c', etc.” differ significantly at p < .05

Covariate		Hispanic (n = 71)	Non-Hispanic American Indian or Alaska Native (n = 1)	Non-Hispanic Asian (n = 22)	Non-Hispanic Black or African American (n = 59)	Non-Hispanic Native Hawaiian or Pacific Islander (n = 6)	Non-Hispanic White (n = 237)	p
Age, median (IQR), years		31 (26.0-40.0)	31 (31.0-31.0)	26 (22.8-36.8)	32 (27.0-36.0)	29.5 (23.3-32.3)	31 (27.0-39.0)	.394
BMI, median (IQR), kg/m^2^		27.8 (23.6-30.4)	23.4 (23.4-23.4)	23.5 (21.0-25.8)	27.5 (23.5-32.0)	26 (22.1-31.0)	24.8 (22.0-28.7)	.003
Operating time, median (IQR), minutes		300 (208.0-367.0)	464 (464.0-464.0)	264 (106.5-323.3)	198 (130.0-291.0)	277.5 (144.5-387.5)	288 (124.0-408.0)	.012
Surgical specialty	General surgery	0 (0.0)_a_	0 (0.0)_a_	0 (0.0)_a_	0 (0.0)_a_	0 (0.0)_a_	1 (0.4)_a_	< .001
	Otolaryngology (ENT)	54 (76.1)_a_	1 (100.0)_ab_	21 (95.5)_a_	21 (35.6)_b_	5 (83.3)_ab_	178 (75.1)_a_	
	Plastic surgery	17 (23.9)_a_	0 (0.0)_ab_	1 (4.5)_a_	38 (64.4)_b_	1 (16.7)_ab_	57 (24.1)_a_	
	Urology	0 (0.0)_a_	0 (0.0)_a_	0 (0.0)_a_	0 (0.0)_a_	0 (0.0)_a_	1 (0.4)_a_	
Elective surgery		71 (100.0)_a_	1 (100.0)_a_	22 (100.0)_a_	59 (100.0)_a_	6 (100.0)_a_	237 (100.0)_a_	-
Hypertension requiring medication		2 (2.8)_a_	0 (0.0)_a_	0 (0.0)_a_	3 (5.1)_a_	0 (0.0)_a_	8 (3.4)_a_	.844
Diabetes	On insulin	0 (0.0)_a_	0 (0.0)_a_	0 (0.0)_a_	0 (0.0)_a_	0 (0.0)_a_	3 (1.3)_a_	.905
	On oral agents	2 (2.8)_a_	0 (0.0)_a_	0 (0.0)_a_	0 (0.0)_a_	0 (0.0)_a_	6 (2.5)_a_	
Dyspnea	Moderate exertion	30 (42.3)_a_	0 (0.0)_a_	8 (36.4)_a_	17 (28.8)_a_	3 (50.0)_a_	63 (26.6)_a_	.110
Current smoker within 1 year prior to surgery		4 (5.6)_a_	1 (100.0)_b_	1 (4.5)_a_	8 (13.6)_ab_	0 (0.0)_ab_	20 (8.4)_a_	.131
Functional health status prior to surgery		71 (100.0)_a_	1 (100.0)_a_	22 (100.0)_a_	59 (100.0)_a_	6 (100.0)_a_	237 (100.0)_a_	-
History of severe COPD		0 (0.0)_a_	0 (0.0)_a_	0 (0.0)_a_	0 (0.0)_a_	0 (0.0)_a_	0 (0.0)_a_	-
Preoperative dialysis		0 (0.0)_a_	0 (0.0)_a_	0 (0.0)_a_	0 (0.0)_a_	0 (0.0)_a_	0 (0.0)_a_	-
Steroid use for chronic condition		0 (0.0)_a_	0 (0.0)_a_	0 (0.0)_a_	0 (0.0)_a_	0 (0.0)_a_	1 (0.4)_a_	1.0
>10% body weight loss within 6 months prior to surgery		30 (42.3)_a_	0 (0.0)_a_	8 (36.4)_a_	17 (28.8)_a_	3 (50.0)_a_	63 (26.6)_a_	.110
Bleeding disorder		0 (0.0)_a_	0 (0.0)_a_	0 (0.0)_a_	0 (0.0)_a_	0 (0.0)_a_	2 (0.8)_a_	1.0
Transfusion ≥1 units of PRBCs in 72 hours prior to surgery		0 (0.0)_a_	0 (0.0)_a_	0 (0.0)_a_	0 (0.0)_a_	0 (0.0)_a_	0 (0.0)_a_	-
Wound classification	1-Clean	55 (77.5)_a_	1 (100.0)_a_	18 (81.8)_a_	42 (71.2)_a_	5 (83.3)_a_	161 (67.9)_a_	.513
	2-Clean/contaminated	16 (22.5)_a_	0 (0.0)_a_	4 (18.2)_a_	17 (28.8)_a_	1 (16.7)_a_	76 (32.1)_a_	
ASA classification	I	23 (32.4)_a_	0 (0.0)_a_	8 (36.4)_a_	13 (22.0)_a_	3 (50.0)_a_	63 (26.6)_a_	.236
	II	40 (56.3)_a_	1 (100.0)_a_	14 (63.6)_a_	37 (62.7)_a_	3 (50.0)_a_	158 (66.7)_a_	
	III	7 (9.9)_a_	0 (0.0)_a_	0 (0.0)_a_	9 (15.3)_a_	0 (0.0)_a_	16 (6.8)_a_	
	IV	1 (1.4)_a_	0 (0.0)_a_	0 (0.0)_a_	0 (0.0)_a_	0 (0.0)_a_	0 (0.0)_a_	

Postoperative complications

Perioperative and 30-day postoperative complications were compared across racial and ethnic groups, stratified by type of GAS. For each complication, multivariable logistic regression was performed to adjust for potential confounders, which were identified based on covariates that had a p < 0.2 upon comparison.

Bivariate analysis of postoperative complications following chest masculinization surgery (Table 9) revealed that Black individuals were significantly more likely to experience unplanned reoperation (n = 14, 4.5%; n = 28, 1.7%; p = 0.042) and unplanned readmission (n = 7, 2.3%; n = 8 0.5%; p = 0.048) compared to White individuals. These differences were not significant in multivariable analysis (Table 10). The frequency of wound disruption (n = 2, 0.1%) and SSI (n = 22, 0.9%) remained low, with no significant differences across racial and ethnic groups. Table 11 shows no significant differences in complications across racial and ethnic groups following chest feminization surgery. Multivariable analysis confirmed the absence of disparities across groups (Table 12).

**Table 9:** Patients’ postoperative complications following chest masculinization surgery across racial and ethnic groups SSI: surgical site infection; DVT: deep vein thrombosis; OR: operating room; *C. diff*: *Clostridioides difficile*; CVA: cerebrovascular accident Frequency data are reported as No. (%). P-values were calculated using Fisher’s exact test due to low expected cell counts across groups. Racial and ethnic categories that do not share the same subscript letter “'a', 'b', 'c', etc.” differ significantly at p < .05

Outcome	Racial and ethnic categories						Total	p
	Hispanic (n = 276)	Non-Hispanic American Indian or Alaska Native (n = 14)	Non-Hispanic Asian (n = 124)	Non-Hispanic Black or African American (n = 311)	Non-Hispanic Native Hawaiian or Pacific Islander (n = 9)	Non-Hispanic White (n = 1661)		
Wound disruption	1 (0.4)_a_	0 (0.0)_a_	0 (0.0)_a_	0 (0.0)_a_	0 (0.0)_a_	1 (0.1)_a_	2 (0.1)	.339
SSI	5 (1.8)_a_	0 (0.0)_a_	0 (0.0)_a_	1 (0.3)_a_	0 (0.0)_a_	16 (1.0)_a_	22 (0.9)	.399
Occurrences pneumonia	0 (0.0)_a_	0 (0.0)_a_	0 (0.0)_a_	0 (0.0)_a_	0 (0.0)_a_	0 (0.0)_a_	0 (0.0)	-
Unplanned intubation	0 (0.0)_a_	0 (0.0)_a_	0 (0.0)_a_	0 (0.0)_a_	0 (0.0)_a_	0 (0.0)_a_	0 (0.0)	-
Pulmonary embolism	0 (0.0)_a_	0 (0.0)_a_	0 (0.0)_a_	0 (0.0)_a_	0 (0.0)_a_	0 (0.0)_a_	0 (0.0)	-
Occurrences ventilator > 48 Hours	0 (0.0)_a_	0 (0.0)_a_	0 (0.0)_a_	0 (0.0)_a_	0 (0.0)_a_	0 (0.0)_a_	0 (0.0)	-
Acute renal failure	0 (0.0)_a_	0 (0.0)_a_	0 (0.0)_a_	0 (0.0)_a_	0 (0.0)_a_	0 (0.0)_a_	0 (0.0)	-
Urinary tract infection	0 (0.0)_a_	0 (0.0)_a_	0 (0.0)_a_	1 (0.3)_a_	0 (0.0)_a_	5 (0.3)_a_	6 (0.3)	1.0
CVA	0 (0.0)_a_	0 (0.0)_a_	0 (0.0)_a_	0 (0.0)_a_	0 (0.0)_a_	0 (0.0)_a_	0 (0.0)	-
Cardiac arrest requiring CPR	0 (0.0)_a_	0 (0.0)_a_	0 (0.0)_a_	0 (0.0)_a_	0 (0.0)_a_	0 (0.0)_a_	0 (0.0)	-
Myocardial infarction	0 (0.0)_a_	0 (0.0)_a_	0 (0.0)_a_	0 (0.0)_a_	0 (0.0)_a_	0 (0.0)_a_	0 (0.0)	-
Bleeding transfusions	0 (0.0)_a_	0 (0.0)_a_	0 (0.0)_a_	1 (0.3)_a_	0 (0.0)_a_	2 (0.1)_a_	3 (0.1)	.667
DVT/thrombophlebitis	0 (0.0)_a_	0 (0.0)_a_	0 (0.0)_a_	0 (0.0)_a_	0 (0.0)_a_	1 (0.1)_a_	1 (0.0)	1.0
Sepsis	0 (0.0)_a_	0 (0.0)_a_	0 (0.0)_a_	1 (0.3)_a_	0 (0.0)_a_	1 (0.1)_a_	2 (0.1)	.519
Septic shock	0 (0.0)_a_	0 (0.0)_a_	0 (0.0)_a_	0 (0.0)_a_	0 (0.0)_a_	0 (0.0)_a_	0 (0.0)	-
Return to OR	9 (3.3)_ab_	0 (0.0)_ab_	4 (3.2)_ab_	14 (4.5)_b_	0 (0.0)_ab_	28 (1.7)_a_	55 (2.3)	.042
Still in hospital > 30 days	0 (0.0)_a_	0 (0.0)_a_	0 (0.0)_a_	0 (0.0)_a_	0 (0.0)_a_	0 (0.0)_a_	0 (0.0)	-
Unplanned readmission	1 (0.4)_ab_	0 (0.0)_ab_	1 (0.8)_ab_	7 (2.3)_b_	0 (0.0)_ab_	8 (0.5)_a_	17 (0.7)	.048
*C. diff* colitis	2 (0.7)_a_	0 (0.0)_a_	2 (1.6)_a_	9 (2.9)_a_	0 (0.0)_a_	46 (2.8)_a_	59 (2.5)	.359

**Table 10:** Multivariate logistic regression for the incidence of complications following chest masculinization surgery SSI: surgical site infection; PE: pulmonary embolism; progressive renal insuff: progressive renal insufficiency; UTI: urinary tract infection; bleeding + transfusion: bleeding requiring transfusion; DVT: deep venous thrombosis; LOS: total length of hospital stay; *C. diff*: *Clostridioides difficile*; BMI: body mass index; aOR: adjusted odds ratio

Outcome		Wound disruption	SSI	Pneumonia	PE	UTI	Bleeding + transfusion	DVT	Sepsis	Unplanned reoperation	LOS >30 days	Unplanned readmission	*C. diff* colitis
		aOR (95% CI)	aOR (95% CI)	aOR (95% CI)	aOR (95% CI)	aOR (95% CI)	aOR (95% CI)	aOR (95% CI)	aOR (95% CI)	aOR (95% CI)	aOR (95% CI)	aOR (95% CI)	aOR (95% CI)
Age		0.9 (0.71-1.23)	1.0 (1.00-1.08)	-	-	1.0 (0.90-1.10)	0.9 (0.8-1.1)	1.0 (0.88-1.23)	0.83 (0.57-1.21)	1.0 (0.94-1.02)	-	1.0 (0.94-1.06)	1.0 (1.0-1.02)
BMI		0.8 (0.56-1.17)	1.0 (0.99-1.10)	-	-	1.1 (1.03-1.20)	1.1 (1.02-1.25)	0.9 (0.68-1.34)	1.0 (0.82-1.24)	1.0 (0.97-1.05)	-	1.1 (1.02-1.14)	1.0 (0.94-1.01)
Total operation time		1.0 (0.98-1.0)	1.0 (1.00-1.01)	-	-	1.0 1.0-1.02	1.0 (0.98-1.02)	1.0 (0.96-1.04)	1.0 (0.97-1.02)	1.0 (0.99-1.0)		1.0 (1.0-1.01)	1.0 (1.0-1.01)
Smoking		10.2 (0.54- >100)	1.0 (0.30-3.52)	-	-	-	-	-	-	0.5 (0.16-1.26)	-	0.8 (0.17-3.39)	1.9 (1.02-3.69)
Hypertension		1.0 -	0.5 (0.06-4.38)	-	-	-	4.5 (0.24-87.07)	-	-	1.0 (0.22-4.81)	-	0.7 (0.08-6.22)	0.5 (0.11-2.23)
Racial and ethnic categories	Non-Hispanic American Indian or Alaska Native	1.0 -	-	-	-	1.1 -	0.9 -	1.3 -	0.9 -	0.0 -	-	0.0 -	0.0 -
	Non-Hispanic Asian	1.0 -	-	-	-	1.5 -	1.8 -	0.9 -	1.0 -	1.0 (0.30-3.28)	-	2.9 (0.18-47.04)	2.4 (0.34-17.75)
	Non-Hispanic Black or African American	1.0 -	0.2 (0.02-1.36)	-	-	>100 -	>100 -	1.1 -	>100 -	1.5 (0.64-3.59)	-	6.2 (0.75-51.28)	4.0 (0.9-18.91)
	Non-Hispanic Native Hawaiian or Pacific Islander	1.0 -	-	-	-	2.6 -	2.0 -	1.1 -	0.8 -	0.0 -	-	0.0 -	0.0 -
	Non-Hispanic White	0.2 (0.0-2.28)	0.5 (0.19-1.44)	-	-	>100 -	>100 -	>100 -	>100 -	0.5 (0.23-1.07)	-	1.3 (0.16-10.75)	4.4 (1.04-18.18)
Constant		2.4	0.001	-	-	0.0	0.0	0.0	0.0	0.07	-	0.0	0.001

**Table 11:** Patients’ postoperative complications following chest feminization surgery across racial and ethnic groups SSI: surgical site infection; DVT: deep vein thrombosis; OR: operating room; *C. diff*: *Clostridioides difficile*; CVA: cerebrovascular accident Frequency data are reported as No. (%). P-values were calculated using Fisher’s exact test due to low expected cell counts across groups. Racial and ethnic categories that do not share the same subscript letter “'a', 'b', 'c', etc.” differ significantly at p < .05

Outcome	Racial and ethnic categories						Total	p
	Hispanic (n = 195)	Non-Hispanic American Indian or Alaska Native (n = 6)	Non-Hispanic Asian (n = 46)	Non-Hispanic Black or African American (n = 195)	Non-Hispanic Native Hawaiian or Pacific Islander (n = 2)	Non-Hispanic White (n = 399)		
Wound disruption	1 (0.5)_a_	0 (0.0)_a_	0 (0.0)_a_	1 (0.5)_a_	0 (0.0)_a_	0 (0.0)_a_	2 (0.2)	.338
SSI	0 (0.0)_a_	0 (0.0)_a_	0 (0.0)_a_	4 (2.1)_a_	0 (0.0)_a_	4 (1.0)_a_	8 (0.9)	.296
Occurrences of pneumonia	0 (0.0)_a_	0 (0.0)_a_	0 (0.0)_a_	0 (0.0)_a_	0 (0.0)_a_	0 (0.0)_a_	0 (0.0)	-
Unplanned intubation	0 (0.0)_a_	0 (0.0)_a_	0 (0.0)_a_	0 (0.0)_a_	0 (0.0)_a_	0 (0.0)_a_	0 (0.0)	-
Pulmonary embolism	0 (0.0)_a_	0 (0.0)_a_	0 (0.0)_a_	0 (0.0)_a_	0 (0.0)_a_	0 (0.0)_a_	0 (0.0)	-
Occurrences ventilator > 48 hours	0 (0.0)_a_	0 (0.0)_a_	0 (0.0)_a_	0 (0.0)_a_	0 (0.0)_a_	0 (0.0)_a_	0 (0.0)	-
Acute renal failure	0 (0.0)_a_	0 (0.0)_a_	0 (0.0)_a_	0 (0.0)_a_	0 (0.0)_a_	0 (0.0)_a_	0 (0.0)	-
Urinary tract infection	2 (1.0)_a_	0 (0.0)_a_	0 (0.0)_a_	1 (0.5)_a_	0 (0.0)_a_	4 (1.0)_a_	7 (0.8)	1.0
CVA	0 (0.0)_a_	0 (0.0)_a_	0 (0.0)_a_	0 (0.0)_a_	0 (0.0)_a_	0 (0.0)_a_	0 (0.0)	-
Cardiac arrest requiring CPR	0 (0.0)_a_	0 (0.0)_a_	0 (0.0)_a_	0 (0.0)_a_	0 (0.0)_a_	0 (0.0)_a_	0 (0.0)	-
Myocardial infarction	0 (0.0)_a_	0 (0.0)_a_	0 (0.0)_a_	0 (0.0)_a_	0 (0.0)_a_	0 (0.0)_a_	0 (0.0)	-
Bleeding transfusions	0 (0.0)_a_	0 (0.0)_a_	0 (0.0)_a_	0 (0.0)_a_	0 (0.0)_a_	0 (0.0)_a_	0 (0.0)	-
DVT/thrombophlebitis	0 (0.0)_a_	0 (0.0)_a_	0 (0.0)_a_	0 (0.0)_a_	0 (0.0)_a_	0 (0.0)_a_	0 (0.0)	-
Sepsis	0 (0.0)_a_	0 (0.0)_a_	0 (0.0)_a_	1 (0.5)_a_	0 (0.0)_a_	1 (0.3)_a_	2 (0.2)	.776
Septic shock	0 (0.0)_a_	0 (0.0)_a_	0 (0.0)_a_	0 (0.0)_a_	0 (0.0)_a_	0 (0.0)_a_	0 (0.0)	-
Return to OR	3 (1.5)_a_	0 (0.0)_a_	0 (0.0)_a_	6 (3.1)_a_	0 (0.0)_a_	3 (0.8)_a_	12 (1.4)	.263
Still in hospital >30 days	0 (0.0)_a_	0 (0.0)_a_	0 (0.0)_a_	0 (0.0)_a_	0 (0.0)_a_	0 (0.0)_a_	0 (0.0)	-
Unplanned readmission	1 (0.5)_a_	0 (0.0)_a_	0 (0.0)_a_	5 (2.6)_a_	0 (0.0)_a_	2 (0.5)_a_	8 (0.9)	.189
*C. diff c*olitis	0 (0.0)_a_	0 (0.0)_a_	0 (0.0)_a_	0 (0.0)_a_	0 (0.0)_a_	8 (2.0)_a_	8 (0.9)	.092

**Table 12:** Multivariate logistic regression for the incidence of complications following chest feminization surgery SSI: surgical site infection; PE: pulmonary embolism; progressive renal insuff: progressive renal insufficiency; UTI: urinary tract infection; bleeding + transfusion: bleeding requiring transfusion; DVT: deep venous thrombosis; LOS: total length of hospital stay; *C. diff*: *Clostridioides difficile*; BMI: body mass index; aOR: adjusted odds ratio

Outcome		Wound disruption	SSI	Pneumonia	PE	UTI	Bleeding + transfusion	DVT	Sepsis	Unplanned reoperation	LOS >30 days	Unplanned readmission	*C. diff* colitis
		aOR (95% CI)	aOR (95% CI)	aOR (95% CI)	aOR (95% CI)	aOR (95% CI)	aOR (95% CI)	aOR (95% CI)	aOR (95% CI)	aOR (95% CI)	aOR(95% CI)	aOR (95% CI)	aOR (95% CI)
Age		-	1.0 (0.95-1.08)	-	-	1.0 (0.93-1.07)	-	-	0.9 (0.57-1.32)	1.0 (1.01-1.13)	-	1.0 (0.97-1.12)	1.0 (0.89-1.03)
BMI		-	0.9 (0.82-1.08)	-	-	1.0 (0.91-1.12)	-	-	1.1 (0.62-2.0)	0.9 (0.79-1.02)	-	0.9 (0.81-1.09)	1.0 (0.82-1.13)
Total operation time		-	1.0 (1.0-1.02)	-	-	1.0 (1.0-1.02)	-	-	1.0 (0.99-1.06)	1.0 (0.99-1.01)	-	1.0 (1.0-1.02)	1.0 (1.0-1.02)
Smoking		-	3.5 (0.67-18.63)	-	-	4.1 (0.81-20.71)	-	-	0.0 -	1.9 (0.51-7.02)	-	2.4 (0.46-12.14)	0.0 -
Hypertension		-	0.9 (0.07-11.71)	-	-	3.0 (0.39-22.63)	-	-	0.0 -	0.0 -	-	0.0 -	1.5 (0.08-29.46)
>10% body weight loss		-	0.5 (0.0- >100)	-	-	6.0 (0.0- >100)	-	-	0.0 -	0.0 -	-	0.0 -	0.6 -
Dyspnea on moderate exertion		-	1.5 (0.0- >100)	-	-	0.5 (0.0- >100)	-	-	0.0 -	>100 -	-	>100 -	0.0 -
Surgical specialty	Orthopedic surgery	-	0.2 -	-	-	>100 -	-	-	>100 -	0.1 -	-	0.1 -	0.0 -
	Plastic surgery	-	>100 -	-	-	>100 -	-	-	>100 -	>100 -	-	>100 -	>100 -
Wound classification	2-Clean/contaminated	-	1.3 (0.0- 17.04)	-	-	0.0 -	-	-	0.0 -	4.2 (0.31-55.49)	-	1.9 (0.11-31.47)	0.7 (0.09-6.27)
	3-Contaminated	-	0.0 -	-	-	0.0 -	-	-	>100 -	0.0 -	-	0.0 -	>100 -
	4-Dirty/infected	-	0.0 -	-	-	0.0 -	-	-	>100 -	>100 -	-	>100 -	>100 -
Racial and ethnic categories	Non-Hispanic American Indian or Alaska Native	-	0.5 -	-	-	0.0 -	-	-	3.3 -	0.0 -	-	0.0 -	1.1 -
	Non-Hispanic Asian	-	0.8 -	-	-	0.0 -	-	-	0.8 -	0.0 -	-	0.0 -	0.6 -
	Non-Hispanic Black or African American	-	>100 -	-	-	0.3 (0.02-4.40)	-	-	0.6 -	2.4 (0.55-10.68)	-	4.1 (0.44-38.93)	0.8 -
	Non-Hispanic Native Hawaiian or Pacific Islander	-	0.5 -	-	-	0.0 -	-	-	>100 -	0.0 -	-	0.0 -	>100 -
	Non-Hispanic White	-	>100 -	-	-	0.7 (0.12-4.71)	-	-	>100 -	0.3 (0.04-1.43)	-	0.5 (0.04-6.6)	>100 -
Constant			0.0	-	-	0.0	-	-	0.0	0.0	-	0.0	0.0

Bivariate analysis of postoperative complications following genital masculinization surgery shows no significant differences across racial and ethnic groups (Table 13). Multivariable analysis confirmed the absence of disparities across groups (Table 14). Among individuals who underwent genital feminization surgery, Hispanic and Black individuals were significantly more likely to experience wound disruption compared with White individuals (n = 12, 9.8%; n = 12, 8.7%; n = 21, 3.3%; p = 0.006; Table 15). Multivariable regression indicated that Non-Hispanic White patients remained at lower odds of wound disruption than Hispanic patients (aOR: 0.5, 95% CI: 0.18-0.84; p < 0.05; Table 16).

**Table 13:** Patients’ postoperative complications following genital masculinization surgery across racial and ethnic groups SSI: surgical site infection; DVT: deep vein thrombosis; OR: operating room; C. diff: *Clostridioides difficile* Frequency data are reported using No. (%). P-values were calculated using Fisher’s exact test due to low expected cell counts across groups. Racial and ethnic categories that do not share the same subscript letter “'a', 'b', 'c', etc.” differ significantly at p < .05

Outcome	Racial and ethnic categories						Total	p
	Hispanic (n = 109)	Non-Hispanic American Indian or Alaska Native (n = 9)	Non-Hispanic Asian (n = 49)	Non-Hispanic Black or African American (n = 150)	Non-Hispanic Native Hawaiian or Pacific Islander (n = 1)	Non-Hispanic White (n = 980)		
Wound disruption	2 (1.8)_a_	0 (0.0)_a_	0 (0.0)_a_	2 (1.3)_a_	0 (0.0)_a_	6 (0.6)_a_	10 (0.8)	.303
SSI	4 (3.7)_a_	0 (0.0)_a_	2(4.1)_a_	4 (2.7)_a_	0 (0.0)_a_	26 (2.7)_a_	36 (2.8)	.773
Occurrences of pneumonia	0 (0.0)_a_	0 (0.0)_a_	0 (0.0)_a_	1 (0.7)_a_	0 (0.0)_a_	2 (0.2)_a_	3 (0.2)	.570
Occurrences of unplanned intubation	0 (0.0)_a_	0 (0.0)_a_	0 (0.0)_a_	1 (0.1)_a_	0 (0.0)_a_	5 (0.1)_a_	6 (0.1)	1.0
Occurrences of pulmonary embolism	0 (0.0)_a_	0 (0.0)_a_	0 (0.0)_a_	0 (0.0)_a_	0 (0.0)_a_	0 (0.0)_a_	0 (0.0)	1.0
Occurrences of ventilator > 48 hours	0 (0.0)_a_	0 (0.0)_a_	0 (0.0)_a_	0 (0.0)_a_	0 (0.0)_a_	1 (0.1)_a_	1 (0.1)	1.0
Occurrences of cerebrovascular accident	0 (0.0)_a_	0 (0.0)_a_	0 (0.0)_a_	0 (0.0)_a_	0 (0.0)_a_	0 (0.0)_a_	0 (0.0)	1.0
Occurrence of myocardial infarction	0 (0.0)_a_	0 (0.0)_a_	0 (0.0)_a_	0 (0.0)_a_	0 (0.0)_a_	0 (0.0)_a_	0 (0.0)	1.0
Acute renal failure	0 (0.0)_a_	0 (0.0)_a_	0 (0.0)_a_	0 (0.0)_a_	0 (0.0)_a_	1 (0.1)_a_	1 (0.1)	1.0
Urinary tract infection	0 (0.0)_a_	0 (0.0)_a_	1 (2.0)_a_	2 (1.3)_a_	0 (0.0)_a_	20 (2.0)_a_	23 (1.8)	.566
Cardiac arrest requiring CPR	0 (0.0)_a_	0 (0.0)_a_	0 (0.0)_a_	0 (0.0)_a_	0 (0.0)_a_	1 (0.1)_a_	1 (0.1)	1.0
Bleeding transfusions	1 (0.9)_a_	0 (0.0)_a_	1 (2.0)_a_	1 (0.7)_a_	0 (0.0)_a_	11 (1.1)_a_	14 (1.1)	.759
DVT/thrombophlebitis	0 (0.0)_a_	0 (0.0)_a_	0 (0.0)_a_	0 (0.0)_a_	0 (0.0)_a_	1 (0.1)_a_	1 (0.1)	1.0
Sepsis	0 (0.0)_a_	0 (0.0)_a_	0 (0.0)_a_	0 (0.0)_a_	0 (0.0)_a_	2 (0.2)_a_	2 (0.2)	1.0
Septic shock	0 (0.0)_a_	0 (0.0)_a_	0 (0.0)_a_	0 (0.0)_a_	0 (0.0)_a_	1 (0.1)_a_	1 (0.1)	1.0
Return to OR	5 (4.6)_a_	0 (0.0)_a_	2 (4.1)_a_	7 (4.7)_a_	0 (0.0)_a_	26 (2.7)_a_	40 (3.1)	.416
Still in hospital > 30 days	0 (0.0)_a_	0 (0.0)_a_	0 (0.0)_a_	1 (0.7)_a_	0 (0.0)_a_	2 (0.2)_a_	3 (0.2)	.570
Unplanned readmission	3 (2.8)_a_	0 (0.0)_a_	0 (0.0)_a_	5 (3.3)_a_	0 (0.0)_a_	23 (2.3)_a_	31 (2.4)	.737
*C. diff* colitis	2 (1.8)_a_	0 (0.0)_a_	4 (8.2)_a_	4 (2.7)_a_	1 (100.0)_a_	34 (3.5)_a_	45 (3.5)	.036

**Table 14:** Multivariate logistic regression for the incidence of complications following genital masculinization surgery SSI: surgical site infection; PE: pulmonary embolism; progressive renal insuff: progressive renal insufficiency; UTI: urinary tract infection; bleeding + transfusion: bleeding requiring transfusion; DVT: deep venous thrombosis; LOS: total length of hospital stay; *C. diff*: *Clostridioides difficile*; ASA: American Society of Anesthesiologists; BMI: body mass index; aOR: adjusted odds ratio

Outcome		Wound disruption	SSI	Pneumonia	PE	UTI	Bleeding + transfusion	DVT	Sepsis	Unplanned reoperation	LOS >30 days	Unplanned readmission	*C. diff* colitis
		aOR (95% CI)	aOR (95% CI)	aOR (95% CI)	aOR (95% CI)	aOR (95% CI)	aOR (95% CI)	aOR (95% CI)	aOR (95% CI)	aOR (95% CI)	aOR(95% CI)	aOR (95% CI)	aOR (95% CI)
BMI		1.0 (0.90-1.012)	1.0 (0.95-1.06)	0.9 (0.75-1.16)	-	1.0 (0.95-1.07)	0.8 (0.74-0.96)	3.8 (0.0- >100)	0.8 (0.34-1.68)	1.0 (0.9-1.01)	1.0 (0.9-1.21)	0.9 (0.86-0.99)	1.0 (0.89-1.02)
Total operation time		1.0 (1.0-1.0)	1.0 (1.0-1.0)	1.0 (1.0-1.01)	-	1.0 (1.0-1.01)	1.0 (1.01-1.02)	1.2 (0.0- >100)	1.0 (1.0-1.05)	1.0 (1.0-1.0)	1.0 (1.0-1.02)	1.0 (1.0-1.01)	1.0 (1.0-1.01)
Smoking		0.9 (0.09-10.34)	1.9 (0.73-4.74)	3.8 (0.18-82.91)	-	1.8 (0.58-5.41)	1.3 (0.12-13.48)	>100 -	1.6 (0.01- >100)	0.7 (0.19-2.27)	0.0 -	1.0 (0.31-3.03)	1.5 (0.56-4.12)
Hypertension		0.6 (0.05-7.33)	0.5 (0.12-1.73)	36.8 (1.45- >100)	-	0.6 (0.13-3.18)	0.6 (0.08-4.34)	0.0 -	19.2 (0.0- >100)	0.6 (0.15-2.11)	5.8 (0.26->100)	1.6 (0.5-5.10)	0.4 (0.05-3.72)
Preoperative dialysis		0.0 -	0.0 -	0.0 -	-	0.7 -	0.0 -	>100 -	>100 -	0.0 -	0.4 -	0.0 -	0.0 -
>10% body weight loss		1.4 (0.17- 11.79)	1.8 (0.51- 6.37)	17.3 (0.0- >100)	-	0.6 (0.09- 3.96)	1.4 (0.26- 7.81)	>100 -	0.7 -	0.9 (0.32-2.48)	>100 -	0.7 (0.15-3.56)	0.0 -
Bleeding disorders		0.0 -	0.0 -	0.0 -	-	0.0 -	0.0 -	>100 -	0.0 -	0.0 -	0.0 -	0.0 -	>100 -
Surgical specialty	Gynecology	>100 -	>100 -	>100 -	-	>100 -	>100 -	0.0 -	>100 -	>100 -	>100 -	>100 -	>100 -
	Obstetric surgery	0.7 -	0.4 -	3.0 -	-	0.4 -	0.1 -	0.0 -	>100 -	0.5 -	2.2 -	1.1 -	1.1 -
	Orthopedic surgery	0.7 -	0.8 -	2.3 -	-	0.4 -	0.2 -	>100 -	0.0 -	0.5 -	0.8 -	0.4 -	0.3 -
	Plastic surgery	>100 -	>100 -	>100 -	-	>100 -	>100 -	0.0 -	0.0 -	>100 -	>100 -	>100 -	>100 -
	Urology	0.7 -	>100 -	0.2 -	-	0.5 -	>100 -	0.0 -	>100 -	>100 -	0.3 -	>100 -	0.9 -
Wound classification	2-Clean/contaminated	2.6 (0.45- 14.77)	1.7 (0.5- 5.77)	14.4 (0.0- >100)	-	1.1 (0.19-5.91)	0.6 (0.12- 3.44)	0.0 -	>100 -	0.8 (0.31-2.11)	>100 -	1.9 (0.48-7.47)	5.3 (0.62- 44.7)
	3-Contaminated	0.0 -	0.0 -	0.0 -	-	0.0 -	0.0 -	>100 -	6.1 -	0.0 -	0.1 -	0.0 -	0.0 -
ASA classification	2-Mild disturbance	1.9 (0.30-12.24)	1.2 (0.48-2.93)	0.2 (0.0-5.61)	-	1.5 (0.46-4.64)	0.4 (0.09-1.97)	0.0 -	>100 -	0.9 (0.42-1.95)	>100 -	2.2 (0.77-6.18)	0.6 (0.27-1.21)
	3-Severe disturbance	3.7 (0.21-67.83)	4.7 (1.32-16.43)	0.4 (0.0-26.09)	-	2.5 (0.45-14.10)	10.9 (1.31-91.31)	>100 -	0.0 -	3.0 (0.82-10.69)	>100 -	3.73 (0.75-18.47)	0.8 (0.15-4.56)
Racial and ethnic categories	Non-Hispanic American Indian or Alaska Native	0.0 -	0.0 -	0.9 -	-	1.1 -	0.0 -	>100 -	0.4 -	0.0 -	0.3 -	0.0 -	0.0 -
	Non-Hispanic Asian	0.0 -	1.0 (0.17-6.21)	0.7 -	-	>100 -	0.5 (0.01-27.39)	0.0 -	2.6 -	0.5 (0.08-3.27)	0.5 -	0.0 -	2.2 (0.29-17.07)
	Non-Hispanic Black or African American	0.4 (0.05-3.11)	0.5 (0.12-2.16)	>100 -	-	>100 -	0.4 (0.02-7.35)	8.2 -	10.5 -	0.7 (0.21-2.47)	>100 -	1.0 (0.23-4.63)	1.7 (0.3-9.67)
	Non-Hispanic Native Hawaiian or Pacific Islander	0.0 -	0.0 -	0.3 -	-	2.9 -	0.0 -	>100 -	>100 -	0.0 -	0.1 -	0.0 -	>100 -
	Non-Hispanic White	0.3 (0.05-1.79)	0.7 (0.24-2.23)	>100 -	-	>100 -	1.6 (0.15-17.19)	0.0 -	>100 -	0.6 (0.2-1.56)	>100 -	0.8 (0.23-2.78)	1.4 (0.32-6.05)
Constant		0.0	0.0	0.0	-	0.0	0.0	-	0.0	0.0	0.0	0.0	0.0

**Table 15:** Patients’ postoperative complications following genital feminization surgery across racial and ethnic groups SSI: surgical site infection; DVT: deep vein thrombosis; OR: operating room; *C. diff.*: *Clostridioides difficile* Frequency data are reported as No. (%). P-values were calculated using Fisher’s exact test due to low expected cell counts across groups. Racial and ethnic categories that do not share the same subscript letter “'a', 'b', 'c', etc.” differ significantly at p < .05

Outcome	Racial and ethnic categories						Total	p
	Hispanic (n = 123)	Non-Hispanic American Indian or Alaska Native (n = 8)	Non-Hispanic Asian (n = 45)	Non-Hispanic Black or African American (n = 138)	Non-Hispanic Native Hawaiian or Pacific Islander (n = 3)	Non-Hispanic White (n = 641)		
Wound disruption	12 (9.8)_a_	0 (0.0)_a,b_	0 (0.0)_a,b_	12(8.7)_a_	0(0.0)_a,b_	21(3.3)_b_	45(4.7)	.006
SSI	3 (2.4)_a_	0 (0.0)_a,b_	1(2.2)_a,b_	7(5.1)_a,b_	1(33.3)_b_	17 (2.7)_a_	29(3.0)	.147
Occurrences of pneumonia	0 (0.0)_a_	0(0.0)_a_	0 (0.0)_a_	0(0.0)_a_	0(0.0)_a_	2 (0.3)_a_	2(0.2)	1.0
Occurrences of unplanned intubation	0 (0.0)_a_	0(0.0)_a_	0 (0.0)_a_	0(0.0)_a_	0(0.0)_a_	1 (0.2)_a_	1(0.1)	1.0
Occurrences of pulmonary embolism	0 (0.0)_a_	0(0.0)_a_	0 (0.0)_a_	1(0.7)_a_	0(0.0)_a_	0 (0.0)_a_	1(0.1)	.331
Occurrences ventilator > 48 hours	0 (0.0)_a_	0(0.0)_a_	0 (0.0)_a_	0(0.0)_a_	0(0.0)_a_	1 (0.1)_a_	1(0.1)	1.0
Occurrences of cerebrovascular accident	0 (0.0)_a_	0(0.0)_a_	0 (0.0)_a_	0(0.0)_a_	0(0.0)_a_	0 (0.0)_a_	0(0.0)	1.0
Occurrences of myocardial infarction	0 (0.0)_a_	0(0.0)_a_	0 (0.0)_a_	0(0.0)_a_	0(0.0)_a_	0 (0.0)_a_	0(0.0)	1.0
Acute renal failure	0 (0.0)_a_	0(0.0)_a_	0 (0.0)_a_	0(0.0)_a_	0(0.0)_a_	1 (0.1)_a_	1(0.1)	1.0
Urinary tract infection	2 (1.6)_a_	0(0.0)_a_	0 (0.0)_a_	3(2.2)_a_	0(0.0)_a_	6 (0.9)_a_	11(1.1)	.549
Cardiac arrest requiring CPR	0 (0.0)_a_	0(0.0)_a_	0 (0.0)_a_	0(0.0)_a_	0(0.0)_a_	1 (0.1)_a_	1(0.1)	1.0
Bleeding requiring transfusion	2 (1.6)_a_	0(0.0)_a_	1 (2.2)_a_	5(3.6)_a_	0(0.0)_a_	10 (1.6)_a_	18(1.9)	.486
DVT/thrombophlebitis	0 (0.0)_a_	0(0.0)_a_	0 (0.0)_a_	1(0.7)_a_	0(0.0)_a_	1 (0.2)_a_	2(0.2)	.553
Sepsis	0 (0.0)_a_	0(0.0)_a_	0 (0.0)_a_	1(0.7)_a_	0(0.0)_a_	3 (0.5)_a_	4(0.4)	.800
Septic shock	0 (0.0)_a_	0(0.0)_a_	0 (0.0)_a_	0(0.0)_a_	0(0.0)_a_	0 (0.0)_a_	0(0.0)	1.0
Return to OR	4 (3.3)_a_	1(12.5)_a_	0(0.0)_a_	7(5.1)_a_	0(0.0)_a_	23 (3.6)_a_	35(3.7)	.370
Still in hospital >30 days	0 (0.0)_a_	0(0.0)_a_	0 (0.0)_a_	0(0.0)_a_	0(0.0)_a_	0 (0.0)_a_	0(0.0)	1.0
Unplanned readmission	4 (3.3)_a_	0(0.0)_a_	1(2.2)_a_	7(5.1)_a_	0(0.0)_a_	21(3.3)_a_	33(3.4)	.830
*C. diff. *colitis	2 (1.6)_a_	2(25.0)_b_	1(2.2)_a,b_	2(1.4)_a_	0(0.0)_a,b_	20 (3.1)_a_	27(2.8)	.076

**Table 16:** Multivariate logistic regression for the incidence of complications following genital feminization surgery SSI: surgical site infection; PE: pulmonary embolism; UTI: urinary tract infection; bleeding + transfusion: bleeding requiring transfusion; DVT: deep venous thrombosis; LOS: total length of hospital stay; *C. diff*: *Clostridioides difficile*; ASA: American Society of Anesthesiologists; BMI: body mass index; aOR: adjusted odds ratio

Outcome		Wound disruption	SSI	Pneumonia	PE	UTI	Bleeding + transfusion	DVT	Sepsis	Unplanned reoperation	LOS >30 days	Unplanned readmission	*C. diff.* colitis
		aOR (95% CI)	aOR (95% CI)	aOR (95% CI)	aOR (95% CI)	aOR (95% CI)	aOR (95% CI)	aOR (95% CI)	aOR (95% CI)	aOR (95% CI)	aOR (95% CI)	aOR (95% CI)	aOR (95% CI)
Age		1.0 (0.99-1.04)	1.0 (1.0-1.06)	0.9 (0.86-1.10)	9.4 (0.0- >100)	1.0 (0.97-1.07)	1.0 (0.91-1.03)	1.1 (1.0-1.24)	1.0 (0.90-1.07)	1.0 (0.99-1.05)	-	1.0 (1.0-1.06)	1.0 (0.98-1.05)
BMI		1.0 (0.95-1.07)	1.0 (0.94-1.08)	1.3 (0.93-1.82)	0.0 (0.0- >100)	1.0 (0.88-1.11)	1.0 (0.85-1.07)	1.0 (0.74-1.45)	1.0 (0.89-1.23)	1.1 (1.0-1.11)	-	1.1 (1.06-1.12)	1.0 (0.97-1.11)
Total operation time		1.0 (1.0-1.01)	1.0 (1.0-1.01)	1.0 (1.0-1.03)	1.2 (0.0- >100)	1.0 (1.0-1.01)	1.0 (1.01-1.02)	1.0 (0.98-1.01)	1.0 (1.0-1.01)	1.0 (1.0-1.01)	-	1.0 (1.0-1.01)	1.0 (0.99-1.0)
Elective surgery		>100 -	>100 -	0.2 -	0.0 -	>100 -	>100 -	0.3 -	1.4 -	>100 -	-	>100 -	>100 -
Smoking		1.8 (0.79-4.23)	3.5 (1.41-8.44)	0.0 -	0.0 -	0.9 (0.10-7.24)	0.5 (0.06-4.21)	0.0 -	0.0 -	1.1 (0.37-3.28)	-	1.1 (0.38-3.36)	2.7 (0.92-8.08)
Hypertension		1.3 (0.47-3.82)	1.1 (0.32-3.86)	0.0 -	0.5 -	3.3 (0.60-17.96)	2.3 (0.30-17.44)	0.0 -	0.0 -	1.4 (0.49-3.76)	-	1.1 (0.40-3.17)	1.2 (0.27-5.09)
Dyspnea	Moderate exertion	0.0 -	>100 -	0.0 -	>100 -	0.0 -	0.0 -	>100 -	0.7 -	0.0 -	-	0.0 -	0.0 -
	At rest	0.0 -	0.0 -	>100 -	>100 -	0.0 -	0.0 -	>100 -	0.9 -	0.0 -	-	0.0 -	>100 -
>10% body weight loss		>100 -	0.0 -	0.0 -	0.0 -	2.0 -	>100 -	0.0 -	0.0 -	>100 -	-	>100 -	>100 -
Bleeding disorders		0.0 -	0.0 -	0.0 -	0.0 -	0.0 -	0.0 -	0.0 -	0.0 -	0.0 -	-	0.0 -	>100 -
Surgical specialty	Gynecology	1.0 -	0.6 -	11.0 -	>100 -	1.2 -	0.0 -	5.9 -	1.8 -	>100 -	-	1.0 -	0.5 -
Otolaryngology (ENT)	2.5 -	1.9 -	56.8 -	>100 -	2.4 -	0.0 -	5.6 -	0.7 -	>100 -	-	2.3 -	1.0 -
Plastic surgery	>100 -	>100 -	>100 -	0.0 -	>100 -	0.1 (0.0-4.60)	>100 -	>100 -	0.3 (0.04-2.69)	-	>100 -	>100 -
Urology	>100 -	>100 -	2.0 -	0.0 -	>100 -	0.0 (0.0-2.47)	0.1 -	>100 -	0.2 (0.03-2.20)	-	>100 -	>100 -
Wound classification	2-Clean/contaminated	1.4 (0.58- 3.34)	2.4 (0.65- 8.81)	>100 -	0.0 -	1.2 (0.24-6.26)	3.4 (0.34- 35.06)	>100 -	1.3 (0.11-14.43)	1.2 (0.48-3.27)	-	0.8 (0.34-2.04)	0.8 (0.27- 2.25)
	3-Contaminated	0.0 -	0.0 -	>100 -	>100 -	0.0 -	0.0 -	16.8 -	0.0 -	0.0 -	-	0.0 -	0.0 -
Racial and ethnic categories	Non-Hispanic American Indian or Alaska Native	0.0 -	0.0 -	8.5 -	>100 -	0.0 -	0.0 -	11.0 -	0.8 -	7.2 (0.63-81.88)	-	0.0 -	17.2 (0.84- >100)
	Non-Hispanic Asian	0.0 -	1.2 (0.12-12.82)	0.3 -	>100 -	0.0 -	1.5 (0.09-26.52)	1.2 -	1.0 -	0.0 -	-	1.0 (0.11-9.70)	5.7 (0.32->100)
	Non-Hispanic Black or African American	0.9 (0.39-2.28)	2.1 (0.5-8.47)	0.1 -	>100 -	1.2 (0.18-7.68)	4.8 (0.54-41.58)	>100 -	>100 -	1.4 (0.40-5.24)	-	1.5 (0.41-5.36)	1.5 (0.13-17.45)
	Non-Hispanic Native Hawaiian or Pacific Islander	0.0 -	28.1 (1.50- >100)	>100 -	>100 -	0.0 -	0.0 -	>100 -	1.2 -	0.0 -	-	0.0 -	0.0 -
	Non-Hispanic White	0.5 (0.18-0.84)	1.1 (0.31-4.10)	>100 -	0.0 -	0.6 (0.11-3.19)	1.8 (0.24-14.16)	>100 -	>100 -	0.6 (0.40-3.66)	-	1.0 (0.34-3.20)	3.1 (0.39-25.13)
Constant		-	-	0.0	>100	0.0	0.0	0.0	0.0	0.0	0.0	0.0	0.0

As shown in Table 17, there were no significant differences in the frequency of complications following facial feminization surgery across racial and ethnic groups. Multivariable analysis confirmed the absence of disparities across groups (Table 18).

**Table 17:** Patients’ postoperative complications following facial feminization surgery across racial and ethnic groups SSI: surgical site infection; DVT: deep vein thrombosis; OR: operating room; *C. diff*: *Clostridioides difficile* Frequency data are reported as No. (%). P-values were calculated using Fisher’s exact test due to low expected cell counts across groups. Racial and ethnic categories that do not share the same subscript letter “'a', 'b', 'c', etc.” differ significantly at p < .05

Outcome	Racial and ethnic categories						Total	p
	Hispanic (n = 71)	Non-Hispanic American Indian or Alaska Native (n = 1)	Non-Hispanic Asian (n = 22)	Non-Hispanic Black or African American (n = 59)	Non-Hispanic Native Hawaiian or Pacific Islander (n = 6)	Non-Hispanic White (n = 237)		
Wound disruption	0 (0.0)_a_	0 (0.0)_a_	0 (0.0)_a_	0 (0.0)_a_	0 (0.0)_a_	0 (0.0)_a_	0 (0.0)	1.0
SSI	2 (2.8)_a_	0 (0.0)_a_	0 (0.0)_a_	0 (0.0)_a_	0 (0.0)_a_	7 (3.0)_a_	9 (2.3)	.701
Occurrences of pneumonia	0 (0.0)_a_	0 (0.0)_a_	0 (0.0)_a_	0 (0.0)_a_	0 (0.0)_a_	0 (0.0)_a_	0 (0.0)	1.0
Occurrences of unplanned intubation	0 (0.0)_a_	0 (0.0)_a_	0 (0.0)_a_	0 (0.0)_a_	0 (0.0)_a_	0 (0.0)_a_	0 (0.0)	1.0
Occurrences of pulmonary embolism	0 (0.0)_a_	0 (0.0)_a_	0 (0.0)_a_	0 (0.0)_a_	0 (0.0)_a_	1 (0.4)_a_	1 (0.3)	1.0
Occurrences of ventilator >48 hours	0 (0.0)_a_	0 (0.0)_a_	0 (0.0)_a_	0 (0.0)_a_	0 (0.0)_a_	0 (0.0)_a_	0 (0.0)	1.0
Occurrences of cerebrovascular accident	0 (0.0)_a_	0 (0.0)_a_	0 (0.0)_a_	0 (0.0)_a_	0 (0.0)_a_	0 (0.0)_a_	0 (0.0)	1.0
Occurrences of myocardial infarction	0 (0.0)_a_	0 (0.0)_a_	0 (0.0)_a_	0 (0.0)_a_	0 (0.0)_a_	0 (0.0)_a_	0 (0.0)	1.0
Acute renal failure	0 (0.0)_a_	0 (0.0)_a_	0 (0.0)_a_	0 (0.0)_a_	0 (0.0)_a_	0 (0.0)_a_	0 (0.0)	1.0
Urinary tract infection	0 (0.0)_a_	0 (0.0)_a_	0 (0.0)_a_	0 (0.0)_a_	0 (0.0)_a_	1 (0.4)_a_	1 (0.3)	1.0
Cardiac arrest requiring CPR	0 (0.0)_a_	0 (0.0)_a_	0 (0.0)_a_	0 (0.0)_a_	0 (0.0)_a_	0 (0.0)_a_	0 (0.0)	1.0
Bleeding transfusions	0 (0.0)_a_	0 (0.0)_a_	0 (0.0)_a_	0 (0.0)_a_	0 (0.0)_a_	1 (0.4)_a_	1 (0.3)	1.0
DVT/thrombophlebitis	0 (0.0)_a_	0 (0.0)_a_	0 (0.0)_a_	0 (0.0)_a_	0 (0.0)_a_	0 (0.0)_a_	0 (0.0)	1.0
Sepsis	0 (0.0)_a_	0 (0.0)_a_	0 (0.0)_a_	0 (0.0)_a_	0 (0.0)_a_	0 (0.0)_a_	0 (0.0)	1.0
Septic shock	0 (0.0)_a_	0 (0.0)_a_	0 (0.0)_a_	0 (0.0)_a_	0 (0.0)_a_	0 (0.0)_a_	0 (0.0)	1.0
Return to OR	0 (0.0)_a_	0 (0.0)_a_	0 (0.0)_a_	0 (0.0)_a_	0 (0.0)_a_	0 (0.0)_a_	0 (0.0)	1.0
Still in hospital >30 days	0 (0.0)_a_	0 (0.0)_a_	0 (0.0)_a_	0 (0.0)_a_	0 (0.0)_a_	0 (0.0)_a_	0 (0.0)	1.0
Unplanned readmission	0 (0.0)_a_	0 (0.0)_a_	0 (0.0)_a_	0 (0.0)_a_	0 (0.0)_a_	0 (0.0)_a_	0 (0.0)	1.0
*C. diff *colitis	0 (0.0)_a_	0 (0.0)_a_	0 (0.0)_a_	0 (0.0)_a_	0 (0.0)_a_	3 (1.3)_a_	3 (0.8)	1.0

**Table 18:** Multivariate logistic regression for the incidence of complications following facial feminization surgery SSI: surgical site infection; PE: pulmonary embolism; progressive renal insuff: progressive renal insufficiency; UTI: urinary tract infection; bleeding + transfusion: bleeding requiring transfusion; DVT: deep venous thrombosis; LOS: total length of hospital stay; *C. diff*: *Clostridioides difficile*; BMI: body mass index; aOR: adjusted odds ratio

Outcome		Wound disruption	SSI	Pneumonia	PE	UTI	Bleeding + transfusion	DVT	Sepsis	Unplanned reoperation	LOS >30 days	Unplanned readmission	*C. diff* colitis
		aOR (95% CI)	aOR (95% CI)	aOR (95% CI)	aOR (95% CI)	aOR (95% CI)	aOR (95% CI)	aOR (95% CI)	aOR (95% CI)	aOR (95% CI)	aOR (95% CI)	aOR (95% CI)	aOR (95% CI)
BMI		-	1.0 (0.94-1.15)	-	1.1 (0.0- >100)	1.2 (0.95-1.44)	1.1 (0.0- >100)	-	-	-	-	-	1.0 (0.78-1.40)
Total operation time		-	1.0 (1.0-1.01)	-	1.2 (0.0- >100)	1.0 (0.98-1.01)	1.2 (0.0- >100)	-	-	-	-	-	1.0 (0.99-1.01)
Smoking		-	3.6 (0.66-19.17)	-	0.0 -	0.0 -	0.0 -	-	-	-	-	-	19.1 (1.15- >100)
Dyspnea on moderate exertion		-	0.2 (0.03-1.87)	-	0.0 -	0.0 -	0.0 -	-	-	-	-	-	0.0 -
Surgical specialty	Otolaryngology (ENT)	-	>100 -	-	0.0 -	4.3 -	0.0 -	-	-	-	-	-	0.0 -
	Plastic surgery	-	>100 -	-	0.0 -	0.0 -	0.0 -	-	-	-	-	-	11.1 -
	Urology	-	0.1 -	-	0.0 -	0.0 -	0.0 -	-	-	-	-	-	0.0 -
Racial and ethnic categories	Non-Hispanic American Indian or Alaska Native	-	0.0 -	-	>100 -	>100 -	>100 -	-	-	-	-	-	>100 -
	Non-Hispanic Asian	-	0.0 -	-	>100 -	0.6 -	>100 -	-	-	-	-	-	6.6 -
	Non-Hispanic Black or African American	-	0.0 -	-	36.9 -	1.2 -	36.9 -	-	-	-	-	-	0.1 -
	Non-Hispanic Native Hawaiian or Pacific Islander	-	0.0 -	-	0.5 -	2.3 -	0.5 -	-	-	-	-	-	1.5 -
	Non-Hispanic White	-	0.9 (0.18-4.84)	-	0.0 -	>100 -	0.0 -	-	-	-	-	-	>100 -
Constant		-	0.0	-	0.0	0.0	0.0	-	-	-	-	-	0.0

## Discussion

The steady increase in the number of GAS performed in recent years reflects its recognized role in addressing gender dysphoria and improving the quality of life for many TGNB individuals [17]. As access to GAS expands, understanding disparities in surgical access and outcomes is crucial to ensuring equitable care. Racial and ethnic disparities in healthcare are well-documented across numerous surgical specialties, yet their impact within GAS has been understudied [6-8]. This gap in the literature is particularly concerning given the compounded inequities faced by individuals who identify as members of multiple marginalized groups [11]. These individuals often experience worse health outcomes due to the intersecting effects of systemic barriers, discrimination, and limited access to care [11]. To address this gap, we analyzed data from 6013 TGNB individuals who underwent GAS between 2012 and 2021, focusing on how racial and ethnic disparities manifest in GAS utilization and complications across various surgical subtypes. These observed associations represent trends that may inform future studies and guide the development of more robust models to better understand and address these disparities.

Overall, our analysis revealed significant disparities in GAS utilization and postoperative complications among TGNB individuals from different racial and ethnic groups. White individuals were more likely to undergo chest and genital masculinization surgeries than Hispanic and Black individuals. Postoperatively, Black individuals were more likely to experience wound disruption following genital feminization surgery, and Hispanic individuals undergoing the same procedure had higher odds of this complication compared to White individuals. Additionally, Black individuals experienced a higher frequency of unplanned reoperations and readmissions following chest masculinization surgeries, although these associations were not statistically significant after multivariable adjustment, likely due to sample size limitations and small group comparisons after stratification. 

Patient-level factors, including preexisting comorbidities, are closely linked to systemic inequities and likely contribute to disparities in surgical complications [18]. In our study, Black individuals had a higher frequency of hypertension, elevated body mass index (BMI), and smoking history, key predictors of poorer surgical outcomes [19,20]. These findings reflect broader inequities in the distribution of income, education, and employment, which disproportionately impact the health status of racial and ethnic minorities [18]. As a result, marginalized TGNB individuals are more likely to have limited access to resources such as nutritious food, stable housing, reliable transportation, and effective healthcare management, further increasing the risk of complications and limiting access to optimal postoperative care [18]. Our finding of increased wound disruption among Black and Hispanic individuals undergoing genital feminization surgery may reflect the impact of unmanaged comorbidities on wound healing, as hypertension and elevated BMI have been associated with wound complications in plastic surgery [21]. Efforts to address these social determinants of health must accompany clinical interventions to achieve equitable outcomes.

Evidence from prior research suggests that systemic inequities in healthcare infrastructure, particularly in insurance coverage and access to high-volume centers (HVCs), perpetuate disparities in surgical utilization and complications for marginalized TGNB individuals [22]. HVCs are associated with better outcomes, including lower complication rates and shorter hospital stays [22]. These inequities disproportionately affect Black and Hispanic individuals, who are also more likely to rely on Medicaid or be uninsured, further limiting their access to experienced providers [17]. Individuals treated at HVCs are more frequently White, privately insured, and within the highest income quartile, whereas individuals who are Black, Hispanic, or Medicaid-insured have significantly lower odds of receiving care at HVCs [22]. These insurance-related barriers compound inequities in surgical care and likely partly explain the lower frequencies of genital masculinization surgery among Hispanic and Black individuals in our cohort, as these procedures require extensive preoperative evaluations, insurance authorizations, and long-term follow-ups. Addressing these systemic issues through equitable insurance policies and increased access to HVCs could significantly enhance access to care, addressing the underlying barriers limiting marginalized TGNB populations’ ability to undergo necessary procedures.

Implicit bias and discrimination within healthcare settings exacerbate these disparities. Provider-level factors, such as stereotypes or assumptions about TGNB individuals of color, contribute to less thorough preoperative counseling and fewer referrals for complex procedures [23]. This bias deepens mistrust of the healthcare system among TGNB individuals, with higher rates of healthcare avoidance reported by people of color due to fear of mistreatment [11]. Furthermore, medical professionals often face challenges in identifying postsurgical complications in individuals with darker skin, which may contribute to disparities in surgical complications. For instance, several studies have found Black race to be associated with higher rates of postoperative hematomas in head and neck surgeries, often requiring unplanned reoperations [24]. One potential reason for this disparity is that changes in skin color, such as signs of poorer perfusion or ecchymosis, can be harder to discern in patients with darker skin tones, making it more challenging to recognize complications [25]. Additionally, disparities in representation may further contribute to inequities in care. Trilles et al. highlighted that while it has been estimated that 45% of TGNB individuals in the United States identify as people of color, just 29% of photographs and 16% of graphics depict individuals of color in TGNB-related medical literature [11]. This lack of representation may reflect broader systemic inequities that perpetuate implicit biases and reduce providers’ ability to diagnose complications in racial and ethnic minorities [11,24].

Previous studies have demonstrated that social and cultural factors also influence GAS utilization and postoperative complications. Many individuals in marginalized communities face significant challenges in receiving adequate social support due to stigma and discrimination associated with their gender identity [26]. For instance, Black and Hispanic individuals often navigate unique cultural pressures surrounding GAS, especially for visible surgeries like chest masculinization [27]. Our results highlight these cultural influences, demonstrating that Black and Hispanic individuals are less likely to undergo chest masculinization surgery compared to White individuals. These patient populations have the highest rate of hiding their transgender identity, as the internalization of stigma and negative attitudes toward their gender identity can hinder them from seeking care and moving forward with their transition [27,28]. Additionally, individuals from marginalized communities may often have increased postoperative complications due to the lack of social support [27,28]. Our findings of higher readmission frequency among Black individuals following chest masculinization surgeries support the possibility that a lack of robust social networks to assist with postoperative care increases the risk of complications [11]. Social networks provide emotional and practical resources, and their absence disproportionately affects marginalized TGNB individuals [28]. Therefore, interventions that focus on building robust social networks and community support systems are crucial.

Our study advances the field by providing a granular analysis of disparities across specific gender-affirming surgical subtypes. While previous research grouped procedures broadly into masculinizing or feminizing categories, we demonstrate that disparities are not uniform across all GAS procedures and identify disparities that might be obscured in broader analyses. For example, we highlight pronounced disparities in genital feminization surgery, where Hispanic and Black individuals face significantly higher risks of complications. This level of detail pinpoints areas where targeted interventions are most urgently needed, such as enhancing postoperative care for genital feminization surgery, increasing access to HVCs for genital masculinization surgery, and implementing cultural competency training for providers. However, our findings must be interpreted considering the study’s limitations. First, stratifying individuals by surgical subtype resulted in smaller sample sizes for some groups, reducing statistical power and potentially obscuring additional significant associations. Second, the ACS-NSQIP database primarily includes HVCs, which may underestimate disparities that are more pronounced in smaller or lower-resourced practices [29]. Third, the database captures only 30-day postoperative outcomes, precluding an analysis of long-term complications [12]. Finally, the ACS-NSQIP lacks data on social determinants of health and access to care, such as educational attainment, occupation, insurance status, and income, limiting our ability to fully assess the systemic and patient-level factors contributing to disparities [30]. Because this study includes only TGNB individuals who underwent surgery, it cannot assess access disparities among those who did not receive care, and related interpretations remain speculative and literature-based. Moving forward, we plan on addressing these gaps by developing a prospective longitudinal registry to better evaluate chronic complications and long-term surgical and psychosocial outcomes in TGNB individuals undergoing GAS. We also plan on integrating the patient-reported outcome measures in our data collection to better evaluate the role of social determinants of health in postoperative outcomes.

## Conclusions

Our study identifies persistent racial and ethnic disparities in GAS utilization and surgical complications. White individuals were more likely to undergo genital masculinization surgery, while Hispanic and Black individuals faced significant barriers to accessing care and higher postoperative complication rates, particularly for genital feminization and chest masculinization surgeries. By stratifying surgical complications by subtype, we contextualize our findings within broader systemic inequities while also identifying the specific procedures where disparities in complications are most pronounced. These insights offer a framework for more granular analysis of patient outcomes and provide a foundation for targeted interventions to improve access to care, reduce complications, and achieve equitable surgical outcomes for TGNB individuals from marginalized racial and ethnic backgrounds.
